# Low‐Energy Electron‐Induced Dissociation of the Radiosensitizing Agent Sanazole

**DOI:** 10.1002/cplu.202500120

**Published:** 2025-05-08

**Authors:** Farhad Izadi, Masoomeh Mahmoodi‐Darian, Thomas F.M. Luxford, Jaroslav Kočišek, Stephan Denifl, Milan Ončák

**Affiliations:** ^1^ Institut für Ionenphysik und Angewandte Physik Universität Innsbruck Technikerstraße 25 6020 Innsbruck Austria; ^2^ Center for Molecular Biosciences Innsbruck Universität Innsbruck Technikerstraße 25 6020 Innsbruck Austria; ^3^ Department of Dynamics of Molecules and Clusters J. Heyrovský Institute of Physical Chemistry of the Czech Academy of Sciences v.v.i., Dolejškova 3 18223 Prague Czech Republic

**Keywords:** electron attachment, electron‐induced dissociation, radiosensitizer, sanazol

## Abstract

Sanazole is a hypoxic radiosensitizer for which the activation mechanism in cells has been suggested to involve initial reduction. Herein, electron attachment to sanazole under isolated conditions and upon microhydrations is investigated. Employing mass spectrometry supported by quantum chemical calculations, the anion formation mechanism and subsequent fragmentation pathways are examined. In the case of electron attachment to the isolated molecule, predominantly dissociative electron attachment is observed. The most prominent fragment anion, (NTR‐yl)^−^ at m/z 113, is suggested to be formed in an exothermic pathway through a single‐bond dissociation, whereas other intense fragments require structural reorganization. The limited abundance of the parent anion under isolated conditions is altered upon microhydration conditions since in the latter situation only the (microhydrated) parent anion is observed. This result suggests that hydration closes and/or slows down the dissociation process and indicates that for sanazole, the initial mechanism of action in a reductive cell environment may be similar to that of well‐studied nitroimidazole radiosensitizers.

## Graphical abstract

Employing mass spectrometry supported by quantum chemical calculations, electron attachment to gas‐phase and microhydrated sanazole, a hypoxic radiosensitizer, is examined. The results suggest that hydration closes and/or slows down the dissociation process and indicates that the initial mechanism of action in a reductive cell environment may be similar to that of well‐studied nitroimidazole radiosensitizers.

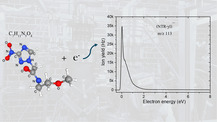

## 1 Introduction

The chemical compound sanazole [AK‐2123, C_7_H_11_N_5_O_4_, N‐(2‐methoxyethyl)‐2‐(3‐nitro‐1,2,4‐triazol‐1‐yl)acetamide, molecular mass 229.193 u] is specifically categorized as a nitrotriazolic radiosensitizer. Sanazole exhibits notable radio‐sensitizing efficacy on hypoxic cells, coupled with a favorable water dissolution rate and low toxicity. Hypoxia, a defining feature of solid tumors, induces tumor cells to develop resistance to ionizing radiation,^[^ ^1^, ^2^ ^]^ whereas the DNA is the primary target for ionizing radiation.^[^ ^1^ ^]^ The prototypes of radiosensitizers mimicking oxygen are nitroimidazoles, and a considerable amount of derivatives were found or synthetized.^[^ ^3^ ^]^ One of the early‐developed compounds was misonidazole, which, however, showed unfavorable results in clinical trials due to dose‐limiting toxicity.^[^ ^4^ ^]^ Nimorazole demonstrated efficacy in several trials and has been recommended for treating head and neck cancers in Denmark, particularly for patients with elevated osteopontin concentrations in their plasma.^[^ ^5^, ^6^ ^]^ Nitrotriazole derivatives have been under investigation as radiosensitizers,^[^ ^7^, ^8^, ^9^ ^]^ and several nitrotriazole derivatives have been used as hypoxia signposts.^[^ ^10^, ^11^, ^12^, ^13^ ^]^ Sanazole has successfully concluded phase‐III clinical trials.^[^ ^14^ ^]^ Pulsed radiolysis experiments with sanazole have revealed its reactivity with aqueous electrons and OH radicals.^[^ ^15^ ^]^ Sanazole showed lower neurotoxicity compared to the majority of nitroimidazoles. This conclusion was drawn from both experimental findings and initial clinical studies.^[^ ^16^ ^]^ Initial trials of sanazole as a radiosensitizer in the irradiation of diverse tumors have shown promising outcomes.^[^ ^7^, ^17^ ^]^ The advantage of employing this compound extends beyond the improvement over radiotherapy alone; it also boasts low toxicity, with no observed potentially fatal hematological effects.^[^ ^9^ ^]^

It was proposed that nitro‐compounds become activated in cells upon initial reduction.^[^ ^18^ ^]^ Thus, a series of studies was carried out with various nitroimidazole molecules, investigating the fundamental process of anion formation upon electron attachment.^[^ ^19^ ^]^ Assuming the situation in the gas phase, a low‐energy electron interacts with a single neutral molecule, AB, which leads to the resonance formation of a temporary negative ion (TNI),

(1) $$e^{-}+AB⇌AB^{*-}$$

The TNI formed in Equation (1) exists in an electronically or vibrationally excited state. Its excess energy includes the initial kinetic energy of the attached electron and the electron affinity of AB. The TNI can relax through autodetachment of the excess electron [indicated by the double‐sided arrow in Equation (1)] or unimolecular decomposition into thermodynamically stable fragments A + B^−^ (known as dissociative electron attachment, DEA). This process was found to form negatively charged fragment ions and neutral radicals even at electron energies considerably below the corresponding bond dissociation energy.^[^ ^20^ ^]^ DEA can be a highly selective process in the cleavage of certain chemical bonds within a molecule.^[^ ^21^ ^]^

The attachment and relaxation processes may vary when transitioning from isolated molecules to clusters, with potential effects, including a reduction in intramolecular fragmentation due to rapid energy distribution or caging effects inhibiting fragment emission.^[^ ^22^ ^]^ These processes may affect the relative abundance of fragment anions, but they may also modify the anion efficiency curve of a specific fragment anion.^[^ ^22^, ^23^, ^24^ ^]^ Even if intramolecular dissociation decreases, the attachment process can remain dissociative, as the dissociation process may be fast along antibonding repulsive σ* states^[^ ^25^ ^]^ or the cluster evaporative cooling may not be enough to remove all the excess energy in the anion for dissociation. On the other hand, this evaporative cooling effect in a sufficiently large cluster can result in the formation of molecular anions, which may go undetected by mass spectrometry in the case of free electron attachment to isolated molecules in the gas phase, such as CO_2_.^[^ ^26^, ^27^ ^]^

In the present work, we delve into the electron attachment‐induced reaction in sanazole, examining two scenarios: Attachment to the isolated molecule [following a recent study with the fundamental 3‐nitro‐1,2,4‐triazole (3NTR) compound using the same experimental setup^[^ ^28^ ^]^] and attachment to the microhydrated molecule within small clusters. The former reveals the fundamental properties of sanazole in low‐energy electron attachment, whereas the latter provides an initial exploration of solvation effects on the formation of TNIs resulting from free electron attachment. To the best of our knowledge, such electron attachment studies with sanazole have not been carried out before.

## 2 Results and Discussion

**Figure 1:**
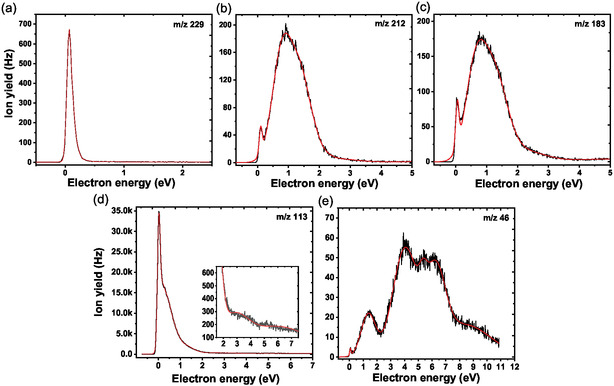
Efficiency curves of anions formed upon electron attachment to sanazole as a function of the electron energy, a) parent anion M^−^ (m/z 229), b) (M‐OH)^−^ (m/z 212), c) (M‐NO_2_)^−^ (m/z 183), d) (NTR‐yl)^−^ (m/z 113), and e) NO_2_^−^ (m/z 46). The black line corresponds to the measured ion yield and the red line to the cumulative fit to the experimental data. Note that the shown electron energy range was chosen by the occurrence of the peaks.

**Figure 2:**
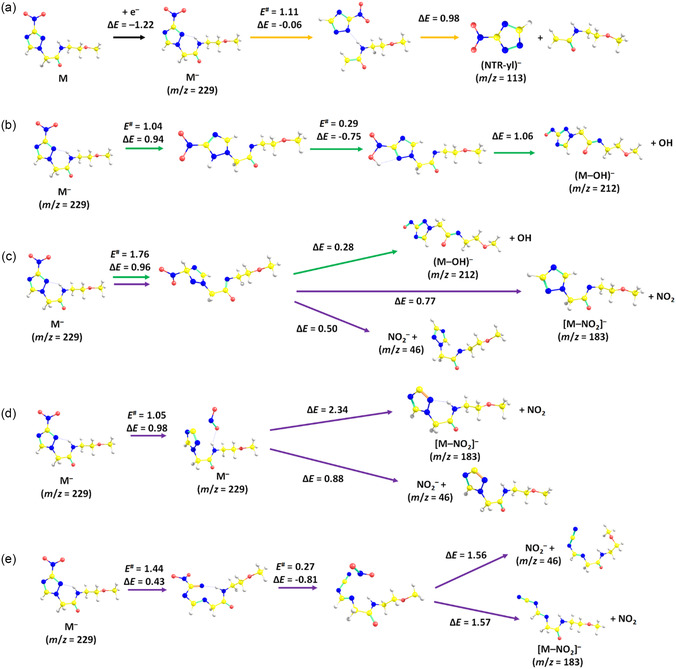
Simplified reaction pathways upon electron attachment to sanazole (M) as calculated at the CCSD/aug‐cc‐pVDZ//*ω*B97XD/aug‐cc‐pVDZ level. Pathways are marked in orange, green, and violet for (NTR‐yl)^−^, (M‐OH)^−^, and (M‐NO_2_)^−^/NO_2_^−^ formation, respectively. Atom color code: yellow – C, blue – N, red – O, white – H.

During the electron attachment experiments with the isolated molecule in Innsbruck, we were able to detect 17 different anionic species. The anion efficiency curves of the five most abundant ones are shown in **Figure** **1**. The peak positions and the onset of the first peak in the anion yields were derived by peak fitting and are listed in **Table** **1** together with the reaction threshold obtained by quantum chemical calculations. The anion efficiency curves of the other 12 fragment anions are included in the Supplementary Material Section, Figures S1–S3, with Table S1, Supporting Information, providing analogous information for these ions as Table 1. Suggested pathways for the production of the most important fragment anions are shown in **Figure** **2** as obtained by quantum chemical calculations.

**Table 1:** Summary of the peak positions and experimental thresholds *E*_thr_ derived from the fits of the anion efficiency curves shown in Figure 1. The calculated reaction energies *E*_calc_ correspond to the dissociation reactions from sanazole + e^−^ producing an anion and a neutral fragment and were obtained at the CCSD/aug‐cc‐pVDZ//*ω*B97XD/aug‐cc‐pVDZ level; see Figure 2 for the respective structures.

Mass (u)	Anion	Peak positions (eV)						*E*_thr_ (eV)	*E*_calc_ (eV)
		1	2	3	4	5	6		
229	M^−^	≈0	–	–	–	–	–	≈0	–1.22
212	(M‐OH)^−^	≈0	0.9	–	–	–	–	≈0	0.03
183	(M‐NO_2_)^−^	≈0	0.8	–	–	–	–	≈0	–0.02
113	(NTR‐yl)^−^	≈0	≈0.3	≈3.6	≈5.4	–	–	≈0	–0.30
46	NO_2_^−^	≈0	1.4	4.0	5.4	6.0	8.2	≈0	–0.04

Among the anions presented in Figure 1, the parent anion of sanazole (m/z 229) is the second most abundant anion observed if the peak maximum is considered. The observation of the parent anion on the timescale of a few hundred microseconds in the Innsbruck experiments indicates that the sanazole molecule has a positive electron affinity and sufficiently withstands autodetachment until detection. However, it is formed just in a single peak at ≈0 eV. At this energy, the parent anion formation is in strong competition with a DEA reaction, as the anion efficiency curve shown in Figure 1d indicates. The vertical and adiabatic electron affinity of sanazole is calculated as 0.54 and 1.22 eV, respectively. Upon electron attachment, the charge is predicted to reside mostly on the NO_2_ group (charge of –0.77 |*e*|), and a hydrogen bond between the N—H bond of the chain and an N atom of the ring is formed (Figure 2a).

The fragment with the highest intensity is (NTR‐yl)^−^ shown in Figure 1d. This fragment anion is formed near 0 eV with about a factor of 40 higher intensity compared to other anions and also reveals an intense shoulder near ≈0.3 eV. The (NTR‐yl)^−^ fragment can be formed from sanazole by simple bond cleavage between the ring moiety and the side chain over a barrier below the entrance channel (i.e., M + e^−^) energy in an overall exothermic reaction (–0.30 eV, see Figure 2a). We note that this is the only fragment ion of Table 1 that is formed in a markedly exothermic reaction. A structurally identical fragment anion was reported as the most abundant fragment anion formed in DEA to 3‐NTR,^[^ ^28^ ^]^ which is formed by H loss from the latter compound. The high abundance of (NTR‐yl)^−^ indicates a high adiabatic electron affinity of the corresponding neutral fragment, here calculated as 4.25 eV. It should be noted that the H abstraction from 3NTR represents an endothermic DEA reaction (calculated reaction threshold ≈+ 0.3 eV), which was also confirmed by the experiment.^[^ ^28^ ^]^ Thus, a weaker bonding situation in sanazole seems to promote the (NTR‐yl)^−^ formation near 0 eV electron energy.

The anion efficiency curves for (M‐OH)^−^ (m/z 212, Figure 1b) and (M‐NO_2_)^−^ (m/z 183, Figure 1c) share a similar shape and intensity. The major peak near 1 eV has about a factor of 3 lower intensity than that of the parent anion formation at 0 eV if the maxima of the peaks are considered. However, accounting for the energy‐integrated ion yields, (M‐OH)^−^ and (M‐NO_2_)^−^ are by about a factor of 3 more abundant than the parent anion. In DEA to 3‐NTR,^[^ ^28^ ^]^ the loss of the OH and NO_2_ fragments were highly abundant DEA channels, having a similar efficiency as the H‐loss. The anion efficiency curves of (3‐NTR‐OH)^−^ and (3‐NTR‐NO_2_)^−^ showed both an abundant peak slightly below 1 eV. Such correlation can be explained by a common precursor resonant state, which decays into different fragment anions with a certain probability. Since presently the same feature can be obtained for sanazole, the involved resonant state seems to be formed by electron attachment to the NTR‐yl moiety. Similar behavior, when the resonant states of the neutral precursor molecules correlate with that of corresponding moieties in larger molecules, has been reported previously.^[^ ^29^, ^30^ ^]^

Our calculations shown in Figure 2 suggest that the common pattern in (M‐OH)^−^ and (M‐NO_2_)^−^ formation at the main peak could be the involvement of the hydrogen atom from the N—H bond. For OH evaporation, the hydrogen to form the OH moiety is predicted to come from the N—H bond, and the respective pathway is shown in Figure 2b. Two barriers must be crossed for hydrogen transfer to a nitrogen atom of the ring and its subsequent transfer to the NO_2_ group, the second barrier lies very close to the entrance channel energy. An alternative pathway, with a hydrogen transfer to the C—NO_2_ ring carbon atom (Figure 2c), requires about 0.5 eV more energy and can be thus operative only at higher energies.

For NO_2_ evaporation, our calculations predict that direct NO_2_ dissociation requires an overall energy of 2.11 eV and is thus impossible near the electron energy of 0 eV (Figure 2d). A considerably lower energy is obtained for the channel with the hydrogen transfer in Figure 2c, but still markedly above the entrance channel energy. An alternative pathway with a transition state only about 0.2 eV above the entrance channel starts with a ring opening, followed by NO_2_ moiety predissociation (Figure 2e). The follow‐up dissociation of NO_2_ results in the reaction energy of –0.02 eV, which is very close to the threshold of (M‐OH)^−^ (0.03 eV). The 0 eV peak found in both anion efficiency curves can be explained by DEA to vibrationally excited sanazole molecules, i.e., a temperature effect on DEA.^[^ ^31^ ^]^ It is interesting to note that the NO_2_ release channel dominates over the simple NO_2_^−^ release or NO release, which are common DEA channels of the nitro compounds.^[^ ^32^, ^33^ ^]^ For ring cleavage, NO_2_ and NO_2_^−^ formation have the same energy within the computational accuracy, suggesting that populating specific electronic states, kinetics, and/or limited accuracy of the calculations could explain the experimentally observed suppression of NO_2_^−^. Overall, the nearly thermoneutral (M‐OH)^−^ and (M‐NO_2_)^−^ formation is induced at electron energies mainly below 2 eV.

The resonant state near 1 eV is also reminiscent of the NO_2_^−^ anion efficiency curve, see Figure 1e. However, the DEA yield peaks at 1.4 eV, i.e., just the higher energy tail of the initial resonant state near 1 eV leads to NO_2_^−^ anion yield. This may also serve as an explanation for why peaks at higher electron energies play a dominating role in the NO_2_^−^ anion efficiency curve, the less abundant peak near 0 eV might be attributed to a hot‐band transition. The peak at 4.0 eV represents a characteristic peak in NO_2_^−^ anion yields from DEA to cyclic molecules with the nitrogen dioxide group, such as, for example, nitroimidazoles. For the fundamental 4‐ and 5‐nitroimidazole isomers (and their partially methylated derivatives), resonances between about 1.5–2 and 3–4 eV were theoretically predicted as the second and third π* shape resonance in these molecular systems.^[^ ^34^ ^]^ Moreover, the authors ^[^ ^34^ ^]^ suggested that rapid electron autodetachment may suppress the occurrence of the DEA peak at lower energy, as experimentally observed for methyl‐nitroimidazoles.^[^ ^35^ ^]^ The π* shape resonance subsequently couples with a certain repulsive σ* state, which may lead to single‐bond cleavage along one specific reaction coordinate. For sanazole, direct NO_2_^−^ formation is calculated to require 0.65 eV of energy and shifts to 0.24 or –0.04 eV when a C—H bond on the ring is formed or ring cleavage takes place, respectively, as discussed above.

Beyond the major peak at 4.0 eV, NO_2_^−^ also abundantly forms at higher electron energies. Two additional peaks at about 5.4 and 6.0 eV have just ≈15% lower intensity compared to the major peak (see Figure 1). Another feature near about 8.2 eV has almost a similar intensity to the peak at ≈1.4 eV. The features at higher electron energies above the 4.0 eV peak may be assigned to core‐excited resonances, where an electron of the molecule is promoted to a formerly unfilled orbital. Such an assignment was also suggested in the theoretical electron scattering study of nitroimidazoles, though electronic excitations were not accounted for in the previous calculations.^[^ ^34^ ^]^ We note that a peak near 6 eV was also present in the NO_2_^−^ anion efficiency curve upon DEA to 3‐NTR and in sanazole the resonance feature may be ascribed to the excitation of the NTR‐yl moiety. On the contrary, the peak at 5.3 eV may be formed with the involvement of the sanazole side chain.

The resonances between about 4 and 9 eV are important also in the formation of other light fragment anions (m/z ≤ 108), as indicated by the anion efficiency curves shown in the supplementary material, Figures S1–S3, Supporting Information. Except for the ((NTR‐yl)‐O)^−^ fragment anion (m/z 97) with the main peak near 1 eV, the peaks at higher electron energies are always the major features for those fragment anions. However, it should be noted that also in the electron energy range between ≈3 and 6 eV, the (NTR‐yl)^−^ anion at m/z 113 represents the most abundant anion formed (see inset in Figure 1d). The similarity of peak energies indicates that these fragment anions share common precursor states of the TNI, which likely represent core‐excited states. At these electron energies, the excess energy deposited into the TNI may also lead to multiple bond ruptures as well as molecular rearrangement in the neutral fragments, which cannot be directly detected in the present experiment.

**Figure 3:**
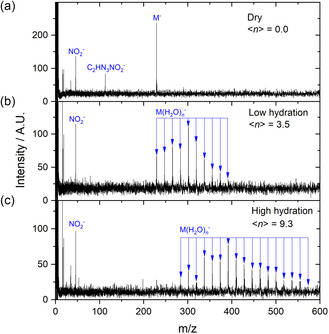
Mass spectra of anions forming upon electron attachment to sanazole measured in CLUB experiment employing molecular beam expansion. The panels represent the sum of mass spectra measured at energies in the 0–9.5 eV range with the step of 0.25 eV. Different expansion conditions result in different neutral M.(H_2_O)_n_ precursors of the attachment reaction, (a) dry conditions *n* = 0, (b) low hydration, and (c) high hydration corresponding to cluster distributions with mean size <n> typically slightly higher than that reported for anions.^[^ ^52^ ^]^

To explore environmental effects on reaction dynamics of sanazole transient negative ions, we performed supporting measurements with microhydrated clusters. The results are shown in **Figure** **3**, which presents cumulative mass spectra of all anions forming upon collisions with 0–9.5 eV electrons. The energy resolution of the experiment is low, not allowing comparison with the high‐resolution spectra for isolated molecules. Still, we present energy‐dependent ion yields for selected fragments in the supporting information. At dry conditions, the mass spectrum in Figure 3 is already dominated by the parent anion, in contrast to the experiment of Innsbruck where the dominant ion is m/z 113 fragment. Two effects cause this difference. First, the temperature of the neutrals in the effusive beam used in Innsbruck is the temperature of sublimation, whereas molecules in the CLUB experiment are cooled to much lower temperatures due to the collisions with Ne atoms during the expansion into the vacuum. The second effect is caused by the different transport times of the used mass analyzers. Although transport times in TOF used in Prague are microseconds, the times for QMS as used in Innsbruck are hundreds of microseconds, which gives the anions more time for decay, as shown in our recent study.^[^ ^36^ ^]^ The suppression of all fragmentation channels with the exception of m/z 113 (the NO_2_^−^ anion yield in Figure 3a can be assigned to the background signal) matches well with our calculations, which suggests that this is the only exothermic channel and the only fragment produced at low energies through direct bond breakage (Figure 2).

Upon hydration, Figure 3b,c, the m/z 113 decay channel is further suppressed and only parent cluster anions, M(H_2_O)_*n*_^−^, and NO_2_^−^ anions can be detected. The NO_2_^−^ anions signal is, however, only due to the background in the experiment. NO_2_^−^ signal is practically constant and dominated by a wide resonance at low energies of electrons, not observed in the case of the Innsbruck experiment (see details in the Supporting Information). We can conclude that in the environment, the sanazole anion will efficiently stabilize similar to other azole derivatives studied in our group.^[^ ^22^, ^37^ ^]^ The stabilized anion then acts as a precursor for multiple electron reduction reactions in the environment.

**Figure 4:**
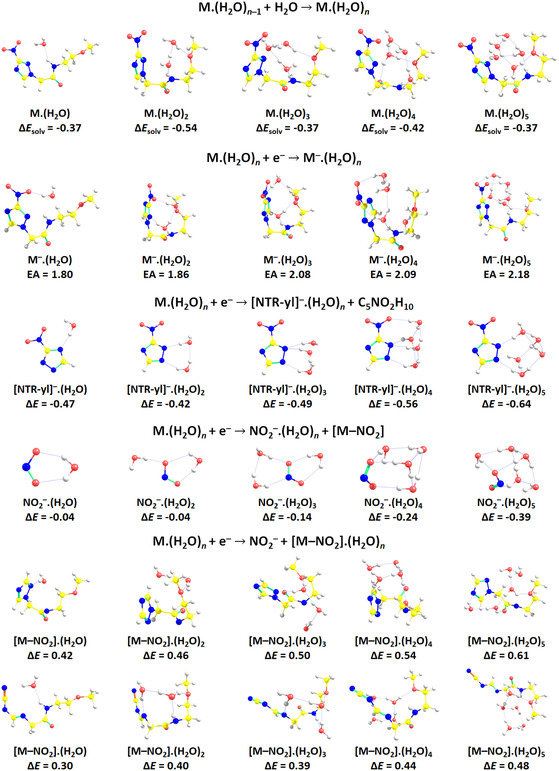
Calculated structures and reaction energies of hydrated sanazole before and upon electron attachment as searched through genetic algorithm optimization and reoptimized at the *ω*B97XD/aug‐cc‐pVDZ level (see Methods for details); the most stable [M–NO_2_] isomer was used for reactions forming NO_2_^−^(H_2_O)_*n*_. Reaction energies are given in eV for the reaction introduced above each set of structures.

To obtain insight into the chemistry of hydrated sanazole, we performed calculations on hydrated M.(H_2_O)_*n*_, *n* = 1–5, as shown in **Figure** **4**. The sanazole molecule is hydrated preferentially on the N—H bond, with an average hydration energy of ≈0.4 eV. The presence of water molecules increases the adiabatic electron affinity up to 2.18 eV for five water molecules (the adiabatic electron affinity of a sanazole molecule is 1.42 eV at *ω*B97XD/aug‐cc‐pVDZ), and a compact structure is formed upon hydration, with the molecule wrapped around the water cluster. Reaction energies for fragments are affected similarly, with the m/z 113 channel being more exothermic by about 0.3 eV for sanazole.(H_2_O)_5_ compared to the dry molecule. NO_2_^−^ formation becomes more exothermic upon solvation due to efficient hydration of the NO_2_^−^ ion. The [M–NO_2_]^−^ ions were considered for both hydrogen transfer and ring cleavage pathway.

The decrease in the reaction enthalpies upon solvation does not agree with the observed suppression of DEA in clusters. There are three possible explanations for that. The first is that the sensitivity of the present experiment is not enough to detect these fragment anions. Sanazole sublimation temperature was kept low to prevent thermal decomposition, and the data were acquired at the detection limits of the CLUB setup. Therefore, we may be selective only to the most intense interaction channel, which is the stabilization of the anion. Another complication was the NO_2_ background, making it hard to disentangle the tiny NO_2_^−^ signal. The second possible explanation is that the formation of NO_2_^−^ anion in cluster results in more complex chemistry resulting in products with low electron affinity, not detectable on the timescale of the experiment. Such behavior can be then illuminated only by experiments detecting neutral DEA products.^[^ ^38^ ^]^ However, based on our previous studies,^[^ ^39^, ^40^ ^]^ we expect at least observation of the (H_2_O)_*n*_.NO_2_^−^ anions, which are very stable. The third explanation is that the calculated reaction thresholds do not describe DEA in the environment, due to significant rearrangement barriers. Particularly in the present case of the clusters cooled down in adiabatic expansion, the compact M.(H_2_O)_*n*_ core can be held by a net of hydrogen bonds, preventing the chemical reactions on the experimental timescale.

## 3 Conclusions

We performed electron attachment experiments on the sanazole molecule in its dry form and upon microsolvation. For the unhydrated molecule, five main channels, including the parent anion, are observed. Our quantum chemical calculations show that the most intense fragment, (NTR‐yl)^−^, is formed through a direct dissociation, whereas other fragments require a hydrogen transfer from the N—H bond to the ring, explaining their lower intensity compared to (NTR‐yl)^−^. Upon microhydration, only the parent anion is observed, suggesting that water molecules stabilize the molecule and prevent bond breaking, with the electron attachment energy most probably dissipated through the evaporation of water molecules. Microhydrated sanazole thus exhibits a very similar characteristic compared to the other well‐known radiosensitizers for hypoxic tumor cells, such as nimorazole and metronidazole. In the case of these two compounds, the stable parent anion dominated the resulting negative ion yields upon electron attachment to the microhydrated compound.^[^ ^22^, ^37^ ^]^ The oxygen‐mimicking effect of the hypoxic cell sensitizer was suggested to rely on initial reduction, followed by the protonation of the parent radical anion, which removes electrons from DNA, thus oxidizing it and causing strand breaks.^[^ ^41^ ^]^ The present results indicate that a reductive pathway by sanazole might get along without the formation of cytotoxic fragment species.

## 4 Experimental Section

### Experiments with Isolated Sanazole

Experimental results for the isolated molecule were obtained by utilizing the crossed electron‐molecular beam instrument in Innsbruck that was previously described in detail in Ref. 28. Here, we provide a brief description of this setup. It consists of a neutral molecular beam source featuring a copper oven with a glass inset as a sample container, a hemispherical electron monochromator (HEM), a quadrupole mass filter (QMA) for the mass analysis of ions, and a channel electron multiplier for ion detection.

The sanazole sample with a sample purity of 90% was purchased at mcule.com Kft., Hungary (supplier Asinex), and used as delivered. The sample was heated in the oven to about 392 K under high vacuum (10^−8^ mbar background pressure). The vapor was guided through a 1 mm diameter capillary into the interaction zone with low‐energy electrons. The HEM was used to generate an electron beam with an energy resolution of ≈120 meV at full width at half maximum (FWHM). The electron current used was in the range of 50–58 nA. In the presence of a weak electrostatic field, anions formed by electron attachment were extracted to the QMA where anions were mass analyzed. The mass‐selected anions were detected by a channeltron secondary electron multiplier operated in the single‐pulse counting mode. The anion efficiency curves were obtained by scanning the electron energy, whereas the QMA was set to transmit a particular anion. For the calibration of electron energy scale and the determination of energy resolution, we utilized the ${\text{Cl}}^{-}$ yield from the well‐known ${\text{CCl}}_{4}$ s‐wave resonance at 0 eV.^[^ ^42^ ^]^ By utilizing the method presented in Ref. 43 , the experimental threshold for the first peak in the recorded anion efficiency curves was derived.

### Experiments on Microhydrated Sanazole

Experiments involving microhydrated sanazole were conducted using the CLUB experimental setup in Prague.^[^ ^44^ ^]^ The experimental configuration closely resembled the one used for studying microhydrated DNA bases.^[^ ^45^ ^]^ A sample of sanazole was sublimed from a glass container, which was placed inside a stainless steel oven. The oven was resistively heated from the exterior wall, where the used temperature (≈433 K) was also monitored using a Pt100 resistance sensor. Sublimation occurred within a neon buffer gas of 5.0 purity, or, in some cases, with a trace amount of water. The mixture of the sample and the buffer gas was passed through a 6 cm long, 6 mm outer diameter Swagelok tube into a 90 μm conical nozzle, through which it was expanded into a vacuum. The tube and nozzle were maintained at a temperature ≈10 °C higher than the oven.

The collisions within the nozzle resulted in a transformation of the random thermal motion of the atoms and molecules in the mixture into a motion in the direction of the nozzle's central axis, forming a supersonic molecular beam. At hydrated conditions, the water–sanazole collisions result in the formation of microhydrated sanazole clusters in the nozzle. The size distribution of such clusters was controlled by adjusting the pressure of the neon buffer gas. ≈1.5 m downstream of the molecular beam, the clusters were collided by electrons from the magnetically collimated electron gun. Products of the electron‐cluster collisions at a specific energy of the incident electrons were m/z analyzed using a reflectron time‐of‐flight mass spectrometer to obtain an anion mass spectrum at the particular electron energy. During the present experiments, 39 spectra at electron energies from 0 to 9.5 eV were acquired at a constant energy step of 0.25 eV and summed up to obtain the cumulative mass spectra, which are presented. The electron current in the present experiment significantly decreases at electron energies below 1.5 eV (see Ref. 46 for details). We account for this decrease by dividing the measured ion yield by electron current at the specific electron energy. Still, in the presented cumulative mass spectra, the intensities of anions forming at electron energies below 1.5 eV could be slightly underestimated.

### Theory

Optimization of neutral and anionic molecules as well as relevant transition states was performed employing density functional (DFT) calculations at the *ω*B97XD/aug‐cc‐pVDZ level. To obtain more reliable reaction energies for the non‐hydrated molecule, single‐point CCSD/aug‐cc‐pVDZ calculations were performed on the optimized structures, using the zero‐point correction as obtained at the *ω*B97XD/aug‐cc‐pVDZ level. Microhydrated sanazole molecules with up to five water molecules were searched for using our in‐house genetic algorithm program, as introduced in detail before^[^ ^47^ ^]^ employing the semi‐empirical method GFN2‐xTB as implemented in the xTB package.^[^ ^48^ ^]^ We used a population of 20 structures with 20 optimization cycles with 10 recombinations per cycle, a mutation probability of 0.2, and fragment preservation.^[^ ^49^ ^]^ Collecting all optimized structures, reoptimizations at the *ω*B97XD/aug‐cc‐pVDZ level were performed on the 12 most stable unique structures for each cluster type; the most stable structure was used for reaction energy calculations. Hydrated fragments formed upon electron attachment were produced from these minima through the removal of the respective molecular parts. Clusters of hydrated NO_2_^−^ were taken from our previous publication.^[^ ^39^ ^]^ Wave function stabilization was performed prior to every DFT calculation. Charge analysis was carried out using the CHELPG scheme.^[^ ^50^ ^]^ The Gaussian software was used for DFT calculations.^[^ ^51^ ^]^

## Conflict of Interest

The author(s) declare no conflict of interest.

## Author Contributions

**Farhad Izadi**: experiments using both setups, data analysis, and cowriting of initial draft. **Masoomeh Mahmoodi‐Darian**: formal analysis, cowriting of initial draft, and editing. **Thomas F.M. Luxford**: experiments using CLUB setup and data analysis. **Jaroslav Kočišek**: experiments using CLUB setup, supervision, cowriting of the initial draft, and editing. **Milan Ončák**: quantum chemical calculations, cowriting of initial draft, and editing. **Stephan Denifl**: conceptualization, supervision, cowriting of initial draft, and editing.

## Supporting information

Supplementary Material

## Data Availability

The data that support the findings of this study are available in the supplementary material of this article.

## References

[1] S. P. Jackson , J. Bartek , Nature 2009, 461, 1071. 19847258 10.1038/nature08467 PMC2906700

[2] M. Rami , L. Dubois , N.-K. Parvathaneni , V. Alterio , S. J. A. van Kuijk , S. M. Monti , P. Lambin , G. de Simone , C. T. Supuran , J.-Y. Winum , J. Med. Chem. 2013, 56, 8512. 24128000 10.1021/jm4009532

[3] G. S. Higgins , S. M. O’Cathail , R. J. Muschel , W. G. McKenna , Cancer Treat. Rev. 2015, 41, 105. 25579753 10.1016/j.ctrv.2014.12.012

[4] S. Dische , M. I. saunders , I. R. Flockhart , M. E. Lee , P. Anderson , Int. J. Radiat. Oncol. Biol. Phys. 1979, 5, 851. 227822 10.1016/0360-3016(79)90070-1

[5] J. Overgaard , H. S. Hansen , M. Overgaard , L. Bastholt , A. Berthelsen , L. Specht , B. Lindeløv , K. Jørgensen , Radiother. Oncol. 1998, 46, 135. 9510041 10.1016/s0167-8140(97)00220-x

[6] J. Overgaard , J. G. Eriksen , M. Nordsmark , J. Alsner , M. R. Horsman , Lancet Oncol. 2005, 6, 757. 16198981 10.1016/S1470-2045(05)70292-8

[7] N. G. Huilgol , N. Chatterjee , A. R. Mehta , Int. J. Radiat. Oncol. Biol. Phys. 1996, 34, 1121. 8600096 10.1016/0360-3016(95)02181-7

[8] Y. S. Chikao Sugie , H. O. Masato Ito , Y. U. Hiromasa Suzuki , H. Nagasawa , H. Hori , J. Radiat. Res. 2005, 46, 453. 16394636 10.1269/jrr.46.453

[9] W. Dobrowsky , N. G. Huigol , R. S. Jayatilake , N.-I.-A. Kizilbash , S. Okkan , V. T. Kagiya , H. Tatsuzaki , Radiother. Oncol. 2007, 82, 24. 17161478 10.1016/j.radonc.2006.11.007

[10] S. Murugesan , S. J. Shetty , O. P. D. Noronha , A. M. Samuel , T. S. Srivastava , C. K. K. Nair , L. Kothari , Appl. Radiat. Isot. 2001, 54, 81. 11144256 10.1016/s0969-8043(00)00104-4

[11] T. Das , S. Chakraborty , S. Banerjee , A. Mukherjee , G. Samuel , H. D. Sarma , C. K. K. Nair , V. T. Kagiya , M. Venkatesh , Bioorg. Med. Chem. 2004, 12, 6077. 15519153 10.1016/j.bmc.2004.09.007

[12] Y. Zhang , T. Chu , X. Gao , X. Liu , Z. Yang , Z. Guo , X. Wang , Bioorg. Med. Chem. Lett. 2006, 16, 1831. 16460938 10.1016/j.bmcl.2006.01.001

[13] R. Bejot , V. Kersemans , C. Kelly , L. Carroll , R. C. King , V. Gouverneur , A. M. Elizarov , C. Ball , J. Zhang , R. Miraghaie , H. C. Kolb , S. Smart , S. Hill , Nucl. Med. Biol. 2010, 37, 565. 20610161 10.1016/j.nucmedbio.2010.03.011

[14] R. Rajagopalan , T. V. Kagiya , C. Krishnan Nair , R. Sanazole , J. Radiat. Res. 2003, 44, 359. 15031563 10.1269/jrr.44.359

[15] R. Mathew , S. Kapoor , C. K. Nair , M. S. Sastry , N. G. Huilgol , C. Gopinathan , B. B. Singh , V. T. Kagiya , Radiat. Phys. Chem. 1997, 49, 233.

[16] Y. Shibamoto , K. Sakano , R. Kimura , T. Nishidai , S. I. Nishimoto , K. Ono , T. Kagiya , M. Abe , Int. J. Radiat. Oncol. Biol. Phys. 1996, 12, 1063. 10.1016/0360-3016(86)90226-9 3744928

[17] V. T. Kagiya , A. H. García‐Angulo , Int. J. Radiat. Oncol. Biol. Phys. 1992, 22, 589. 1735697 10.1016/0360-3016(92)90883-j

[18] G. E. Adams , E. D. Clarke , I. R. Flockhart , R. S. Jacobs , D. S. Sehmi , I. J. Stratford , P. Wardman , M. E. Watts , J. Parrick , R. G. Wallace , C. E. Smithen , INT. J. RADIAT. BIOL. 1979, 35, 133. 10.1080/09553007914550151 312783

[19] B. Sedmidubská , J. Kočišek , Phys. Chem. Chem. Phys. 2024, 26, 9112. 38376461 10.1039/d3cp06003a

[20] I. Baccarelli , I. Bald , F. A. Gianturco , E. Illenberger , J. Kopyra , Phys. Rep. 2011, 508, 1.

[21] S. Ptasinska , S. Denifl , P. Scheier , E. Illenberger , T. D. Märk , Angew. Chem. Int. Ed. 2005, 44, 6941. 10.1002/anie.200502040 16206311

[22] R. Meißner , J. Kočišek , L. Feketeová , J. Fedor , M. Fárník , P. Limão‐Vieira , E. Illenberger , S. Denifl , Nat. Commun. 2019, 10, 2388. 31160602 10.1038/s41467-019-10340-8 PMC6546713

[23] T. D. Märk , Int. J. Mass Spectrom. Ion Processes 1991, 107, 143.

[24] F. W. Oddur Ingolfsson , E. Illenberger , Int. J. Mass Spectrom. Ion Processes 1996, 155, 1.

[25] M. Neustetter , J. Aysina , F. F. Da Silva , S. Denifl , Angew. Chem. Int. Ed. 2015, 54, 9124. 10.1002/anie.201503733 PMC4832840 26110285

[26] S. Denifl , V. Vizcaino , T. D. Märk , E. Illenberger , P. Scheier , Phys. Chem. Chem. Phys. 2010, 12, 5219. 21491691 10.1039/b924526j

[27] S. Matejcik , P. Stampfli , A. Stamatovic , P. Scheier , T. D. Märk , J. Chem. Phys. 1999, 111, 3548.

[28] M. Saqib , F. Izadi , L. U. Isierhienrhien , M. Ončák , S. Denifl , Phys. Chem. Chem. Phys. 2023, 25, 13892. 37183636 10.1039/d3cp01162c PMC10207873

[29] V. S. Prabhudesai , A. H. Kelkar , D. Nandi , E. Krishnakumar , Phys. Rev. Lett. 2005, 95, 143202. 16241651 10.1103/PhysRevLett.95.143202

[30] M. Zawadzki , T. F. M. Luxford , J. Kočišek , J. Phys. Chem. A 2020, 124, 9427. 33125242 10.1021/acs.jpca.0c07283 PMC7667636

[31] M. Mahmoodi‐Darian , A. Mauracher , A. Aleem , S. Denifl , B. Rittenschober , A. Bacher , M. Probst , T. D. Märk , P. Scheier , J. Phys. Chem. A 2009, 113, 14923. 19877656 10.1021/jp9050726

[32] R. Schürmann , T. F. M. Luxford , I. S. Vinklárek , J. Kočišek , M. Zawadzki , I. Bald , J. Chem. Phys. 2020, 153, 104303. 32933272 10.1063/5.0018784

[33] T. F. M. Luxford , S. A. Pshenichnyuk , N. L. Asfandiarov , T. Perečko , M. Falk , J. Kočišek , Int. J. Mol. Sci. 2020, 21, 8173. 33142925 10.3390/ijms21218173 PMC7662275

[34] F. Kossoski , M. T. Varella , J. Chem. Phys. 2017, 147, 164310. 29096502 10.1063/1.5005604

[35] K. Tanzer , L. Feketeová , B. Puschnigg , P. Scheier , E. Illenberger , S. Denifl , Angew. Chem. Int. Ed. 2014, 53, 12240. 10.1002/anie.201407452 PMC4501305 25224248

[36] F. Izadi , T. F. M. Luxford , B. Sedmidubská , E. Arthur‐Baidoo , J. Kočišek , M. Ončák , S. Denifl , Angew. Chem. Int. Ed. 2024, 63, e202407469. 10.1002/anie.202407469 38980970

[37] C. Lochmann , T. F. M. Luxford , S. Makurat , A. Pysanenko , J. Kočišek , J. Rak , S. Denifl , Pharmaceuticals 2022, 15, 701. 35745620 10.3390/ph15060701 PMC9227036

[38] Z. Li , A. R. Milosavljević , I. Carmichael , S. Ptasinska , Phys. Rev. Lett. 2017, 119, 53402. 10.1103/PhysRevLett.119.053402 28949760

[39] J. Lengyel , M. Ončák , J. Fedor , J. Kočišek , A. Pysanenko , M. K. Beyer , M. Fárník , Phys. Chem. Chem. Phys. 2017, 19, 11753. 28397887 10.1039/c7cp01205e PMC5450009

[40] D. Šmídová , J. Lengyel , J. Kočišek , A. Pysanenko , M. Fárník , Int. J. Mass Spectrom. 2017, 421, 144.

[41] D. I. Edwards , J. Antimicrob. Chemother. 1993, 31, 9. 10.1093/jac/31.1.9 8444678

[42] G. A. Gallup , K. Aflatooni , P. D. Burrow , J. Chem. Phys. 2003, 118, 2562.

[43] R. Meißner , L. Feketeová , A. Bayer , J. Postler , P. Limão‐Vieira , S. Denifl , J. Mass Spectrom. 2019, 54, 802. 31410948 10.1002/jms.4427 PMC6916310

[44] M. Fárník , J. Fedor , J. Kočišek , J. Lengyel , E. Pluhařová , V. Poterya , A. Pysanenko , Phys. Chem. Chem. Phys. 2021, 23, 3195. 33524089 10.1039/d0cp06127a

[45] J. Kočišek , A. Pysanenko , M. Fárník , J. Fedor , J. Phys. Chem. Lett. 2016, 7, 3401. 27525662 10.1021/acs.jpclett.6b01601

[46] J. Kočišek , K. Grygoryeva , J. Lengyel , M. Fárník , J. Fedor , Eur. Phys. J. D 2016, 70, 1.

[47] B. Kocábková , G. Schöpfer , J. Ďurana , J. Rakovský , V. Poterya , M. Gatt , D. Jank , M. Ončák , M. Fárník , Phys. Scr. 2024, 99, 125410.

[48] C. Bannwarth , E. Caldeweyher , S. Ehlert , A. Hansen , P. Pracht , J. Seibert , S. Spicher , S. Grimme , WIREs Comput. Mol. Sci. 2021, 11, e1493.

[49] Schöpfer G. , Gatt M. , Ončák M. , Genetic Algorithms, 2024, https://git.uibk.ac.at/c7441332/genetic‐algorithms.

[50] C. M. Breneman , K. B. Wiberg , J. Comput. Chem. 1990, 11, 361.

[51] M. J. Frisch , G. W. Trucks , H. B. Schlegel , G. E. Scuseria , M. A. Robb , J. R. Cheeseman , G. Scalmani , V. Barone , G. A. Petersson , H. Nakatsuji , X. Li , M. Caricato , A. V. Marenich , J. Bloino , B. G. Janesko , R. Gomperts , B. Mennucci , H. P. Hratchian , J. V. Ortiz , A. F. Izmaylov , J. L. Sonnenberg , D. Williams‐Young , F. Ding , F. Lipparini , F. Egidi , J. Goings , B. Peng , A. Petrone , T. Henderson , D. Ranasinghe , et al., Gaussian 16 Revision A.03, 2016.

[52] J. Poštulka , P. Slavíček , J. Fedor , M. Fárník , J. Kočišek , J. Phys. Chem. B 2017, 121, 8965. 28858504 10.1021/acs.jpcb.7b07390

