# Supplementary material for: Low‐Energy Electron‐Induced Dissociation of the Radiosensitizing Agent Sanazole
Source: Chempluschem. 2025 May 8;90(7):e202500120. doi: 10.1002/cplu.202500120 (PMC12261046; doi:10.1002/cplu.202500120)
Supplement: Supplementary file 1 — Supplementary Material [file CPLU-90-e202500120-s001.pdf]

Supporting Information

for

Low-Energy Electron Induced Dissociation of the Radiosensitizing Agent Sanazole

Farhad Izadi[1,2], Masoomeh Mahmoodi-Darian[1], Thomas F.M. Luxford[3], Jaroslav Kočišek*[3], Stephan Denifl,*[1,2] Milan Ončák*[1]

[1] *Institut für Ionenphysik und Angewandte Physik, Universität Innsbruck, Technikerstraße 25, 6020 Innsbruck, Austria.*

[2] *Center for Molecular Biosciences Innsbruck, Universität Innsbruck, Technikerstraße 25, 6020 Innsbruck, Austria.*

[3] *J. Heyrovský Institute of Physical Chemistry of the Czech Academy of Sciences, v.v.i., Dolejškova 3, 18223 Prague, Czech Republic*

** Corresponding authors: jaroslav.kocisek@jh-inst.cas.cz, stephan.denifl@uibk.ac.at, milan.oncak@uibk.ac.at*

Table of Contents:

S1. Additional experimental data……….....……………….……………………………..…..... 2

S2. Cartesian coordinates and electronic energies of optimized structures …………………. 8

S1. Additional experimental data

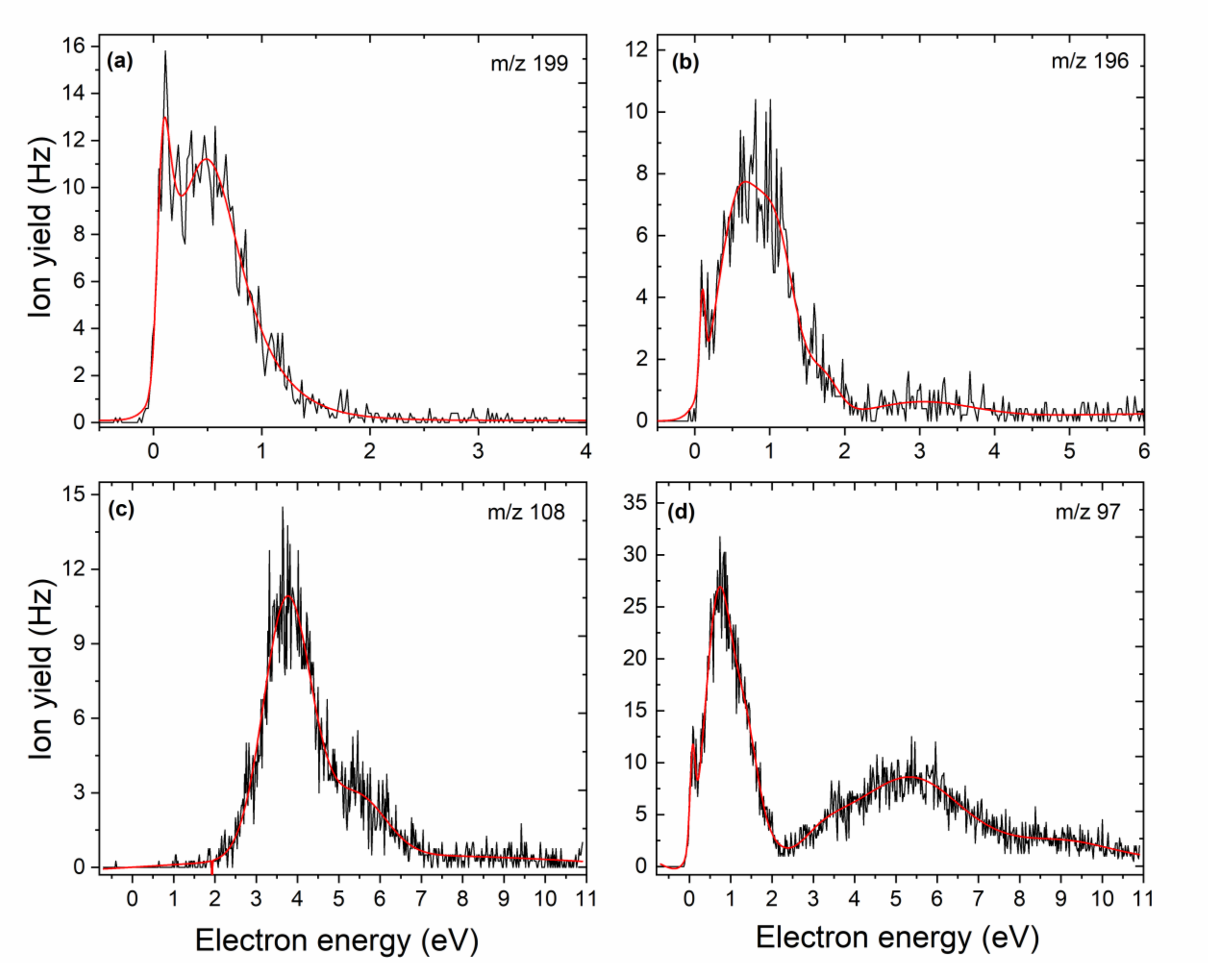

Figure S1. Efficiency curves of anions formed upon electron attachment to sanazole as a function of the electron energy, (a) (M-NO)$^-$ (m/z 199), (b) (M-CH_5O)$^-$ (m/z 196), (c) ($C_4H_2N_3O$)$^-$ (m/z 108), and (d) ((NTR-yl)-O)$^-$ (m/z 97). The black line corresponds to the measured ion yield and the red line to the cumulative fit to the experimental data.

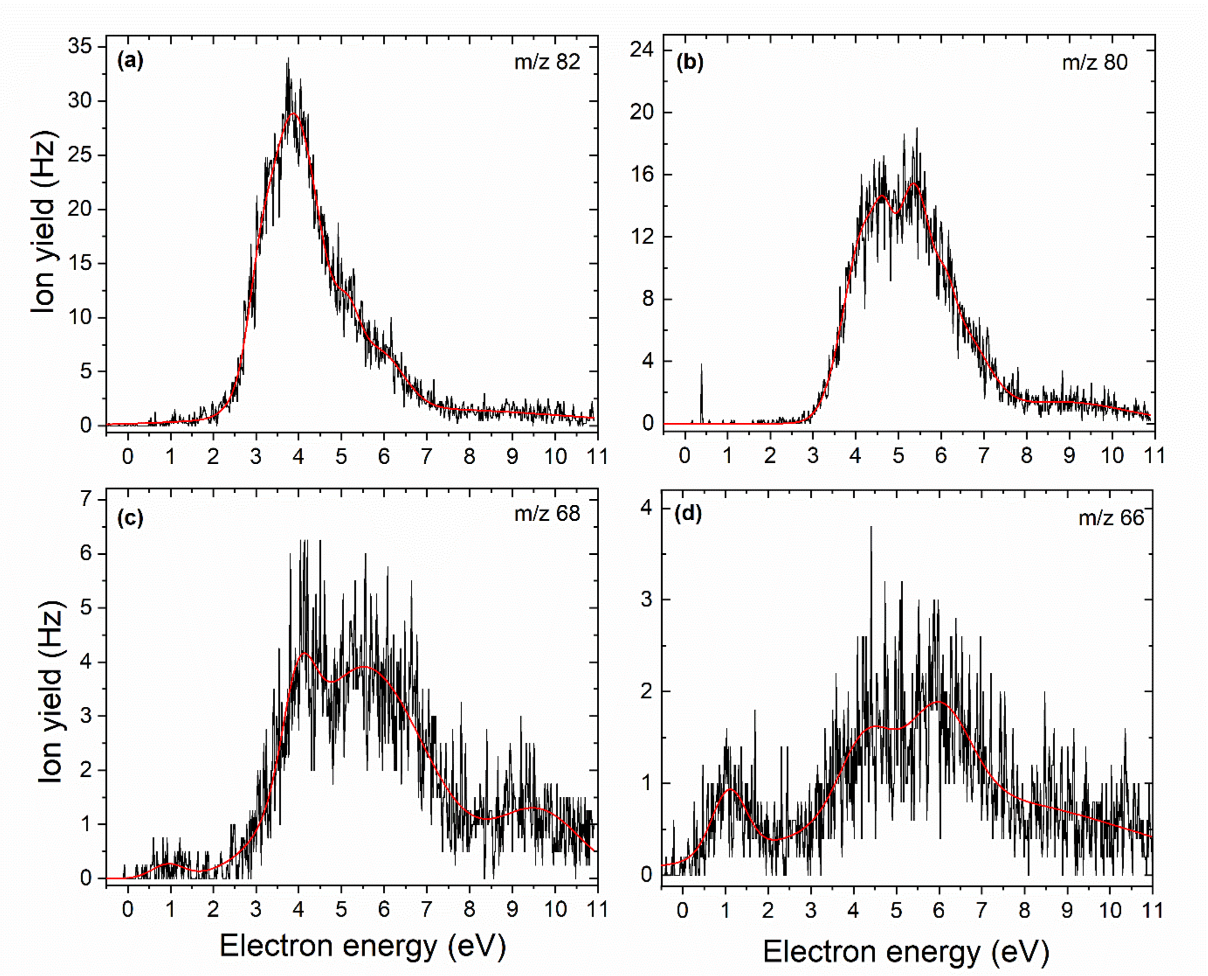

Figure S2. Efficiency curves of anions formed upon electron attachment to sanazole as a function of the electron energy, (a) $(C_2N_3O)^-$ (m/z 82), (b) $(C_3H_2N_3)^-$ (m/z 80), (c) $(C_2N_2O)^-$ (m/z 68), and (d) $(C_2N_3)^-$ (m/z 66). The black line corresponds to the measured ion yield and the red line to the cumulative fit to the experimental data. The fits were derived using an exponentially modified Gaussian peak function.

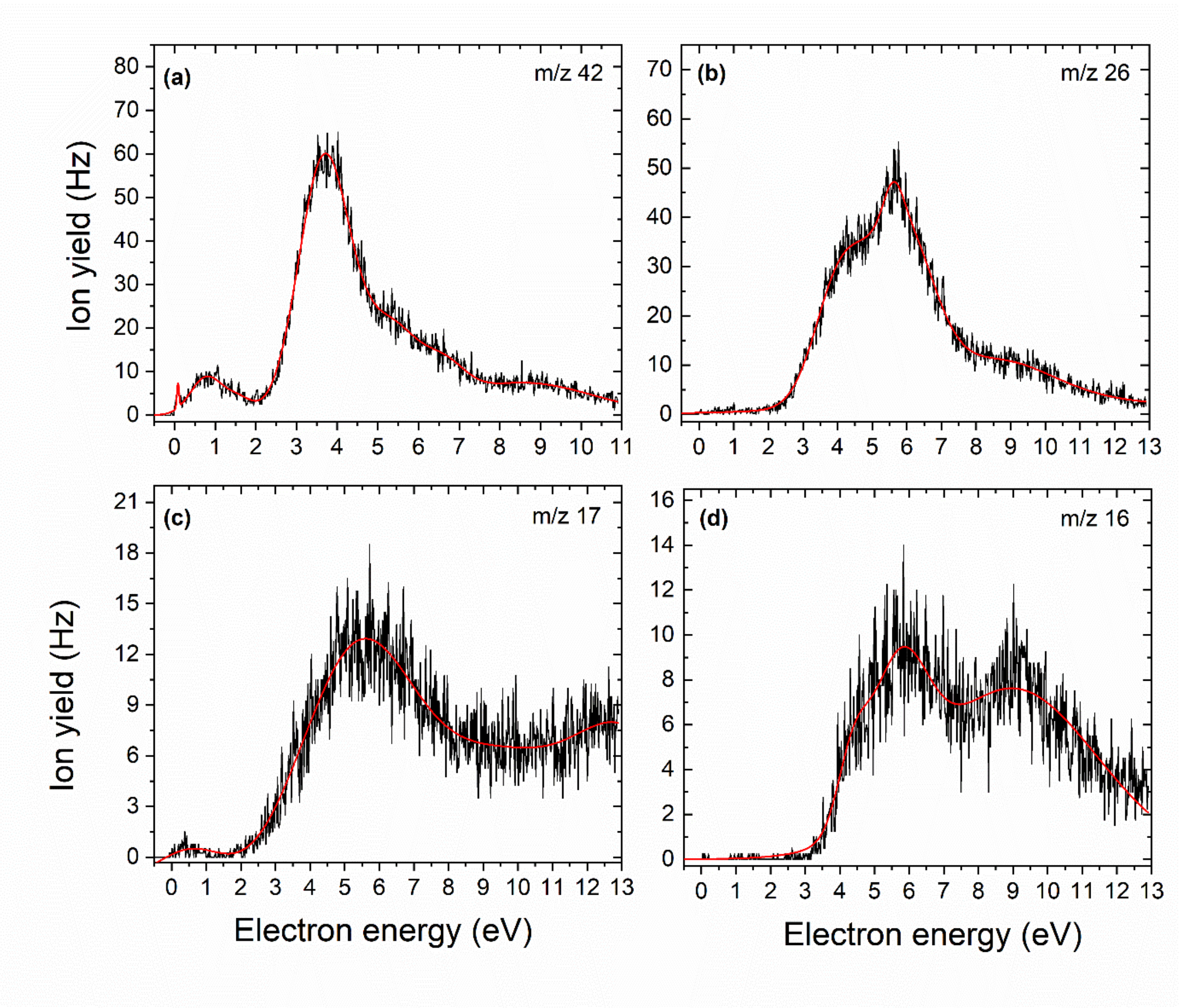

Figure S3. Efficiency curves of anions formed upon electron attachment to sanazole as a function of the electron energy, (a) $(C_2H_4N)^-/CNO^-$ (m/z 42), (b) CN^- (m/z 26), (c) OH^- (m/z 17), and (d) O^- (m/z 16). The black line corresponds to the measured ion yield and the red line to the cumulative fit to the experimental data.

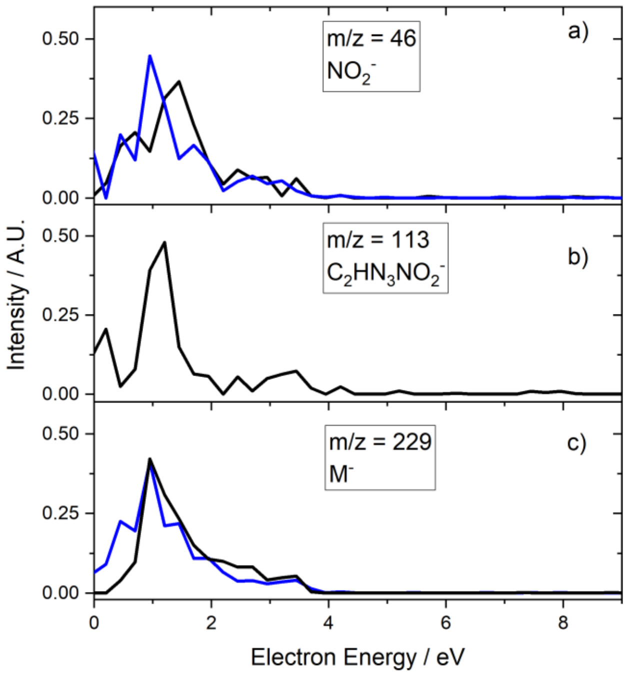

Figure S4. Efficiency curves of anions formed upon electron attachment to sanazole and its clusters as a function of the electron energy, acquired using CLUB experiment in Prague. The black line corresponds to the measured ion yield for "dry" conditions, the blue line for "high hydration" conditions as explained in the main text. The dependencies are distorted due to the wide electron energy distribution function and strong decrease of the electron current at energies below 1 eV [see 10.1140/epjd/e2016-70074-0]

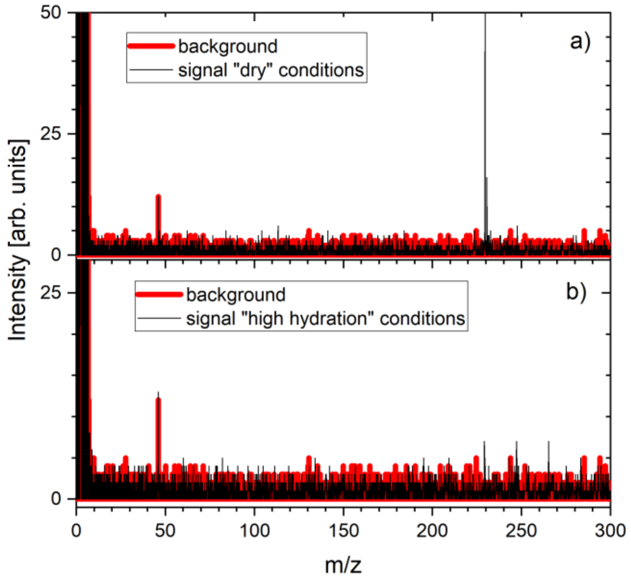

Figure S5. Mass spectra measured at single energy of incident electrons 2 eV. The upper panel a) corresponding to "dry" conditions and the lower panel b) to "high hydration" conditions. The measurements of mass spectra present in the main text took several hours, giving enough time for the variation of the background level. To ensure the NO_2 signal is not influenced by hydration level, we performed this supporting measurement fast changing the expansion conditions between "dry" conditions and "high hydration" conditions as explained in the main text.

Table S1: Summary of the peak positions and experimental thresholds E_{trh} derived from the fits of the anion efficiency curves shown in Figures S1-S3.

Mass (u)	Anion	Peak positions (eV)						
		1	2	3	4	5	6	E_{thr} (eV)
199	$(M\text{-}NO)^-$	~0	~0.5	--	--	--	--	~0
196	$(M\text{-}CH_5O)^-$	~0	~0.8	~3.0	--	--	--	~0
108	$(C_4H_2N_3O)^-$	3.8	5.5	--	--	--	--	2.5
97	((NTR-yl)-O)$^-$	0	0.8	5.3	~9	--	--	~0
82	$(C_2N_3O)^-$	3.8	~5.1	~5.9	--	--	--	2.4
80	$(C_3H_2N_3)^-$	4.6	5.4	~6.3	~9.0	--	--	3.0
68	$(C_2N_2O)^-$	0.9	4.1	5.5	9.6	--	--	~0.1
66	$(C_2N_3)^-$	1.0	4.3	6.0	--	--	--	0.13
42	$(C_2H_4N)^-/CNO^-$	0	0.8	3.7	5.3	6.7	8.6	~0
26	CN^-	4.3	5.7	9.1	--	--	--	2.3
17	OH^-	~0.5	5.6	--	--	--	--	~0.1
16	O^-	4.3	5.8	9.0	--	--	--	3.3

S2. Cartesian coordinates and energies of optimized structures

(NRT-yl)-
E = -446.087885
N -1.185194 0.688255 0.000000
N -0.880416 1.997806 0.000000
C 0.466667 2.060297 0.000000
N 1.081099 0.871137 0.000000
C 0.000000 0.071413 0.000000
N 0.103859 -1.364780 0.000000
O 1.224295 -1.866019 0.000000
O -0.928839 -2.027200 0.000000
H 1.000912 3.008574 0.000000

(NRT-yl)
E = -445.923279
N 0.801139 -1.065636 -0.437442
N 2.022943 -0.713279 -0.293924
C 2.002766 0.598185 0.240202
N 0.772350 1.076003 0.426827
C 0.062088 0.045012 0.006615
N -1.389423 0.011423 0.006001
O -1.956787 0.824472 -0.690833
O -1.887626 -0.844221 0.708555
H 2.917118 1.139236 0.467093

NO2
E = -204.983361
N -0.000000 0.000000 0.551191
O 0.000000 0.964694 -0.241146
O -0.000000 -0.964694 -0.241146

NO2-
E = -205.114840
N 0.000000 0.000000 0.458583
O -0.000000 1.064885 -0.200630
O -0.000000 -1.064885 -0.200630

OH
E = -75.713284
O 0.000000 0.000000 0.108286
H 0.000000 0.000000 -0.866285

[M-NTR-yl]
E = -401.466274
H -4.375218 0.418802 -0.160931
C -3.446114 0.476984 0.416311
H -3.199949 1.537113 0.600784
H -3.593106 -0.025767 1.388932
O -2.441770 -0.155686 -0.339365
C -1.191099 -0.143881 0.305911
H -0.880235 0.888011 0.544540
H -1.237925 -0.718110 1.251364
C -0.174675 -0.774199 -0.638836
H -0.108008 -0.169561 -1.550922
H -0.501098 -1.784643 -0.913616
N 1.142881 -0.857109 -0.042808
H 1.415840 -1.704838 0.427721
C 2.000212 0.213904 -0.029142
O 1.700913 1.293677 -0.538423
C 3.287508 -0.007018 0.616453
H 3.558036 -0.959161 1.073419
H 3.993362 0.819243 0.636488

M
E = -847.542358
N 1.465923 1.294421 0.332695
C 2.501098 1.736964 -0.411207
N 3.366403 0.761696 -0.624301
C 2.779677 -0.262120 0.015894
N 1.633016 -0.006730 0.615887
H 2.582696 2.759218 -0.764310
N 3.356973 -1.604923 0.061154
O 2.646330 -2.490288 0.499506
O 4.494071 -1.725502 -0.343406
C 0.283106 2.012493 0.773143
H 0.173441 1.871594 1.855286
H 0.437825 3.076106 0.572447
C -1.031028 1.610384 0.088638
N -1.146620 0.321504 -0.295885
O -1.900051 2.457052 -0.045460
C -2.366604 -0.188984 -0.896494
H -0.409614 -0.325035 -0.044266
C -3.381137 -0.607527 0.159969
H -2.797949 0.595436 -1.527796
H -2.110401 -1.046776 -1.528832
O -4.503289 -1.124629 -0.510093
H -2.940009 -1.371876 0.827778
H -3.657763 0.267814 0.774283
C -5.530429 -1.512001 0.372144
H -6.354366 -1.898390 -0.236923
H -5.188007 -2.303527 1.061649
H -5.890294 -0.654661 0.966939

[M-NO2]
E = -642.400083
N 2.424564 0.200732 0.330823
C 3.504883 0.218514 -0.468725
N 4.047610 -0.993659 -0.551692
C 3.225071 -1.697712 0.223347
N 2.226239 -1.071344 0.796328
H 3.863271 1.118361 -0.958094
C 1.514597 1.276950 0.663096
H 1.383969 1.304507 1.751946
H 1.969587 2.220354 0.346288
C 0.128966 1.203215 0.011172
N -0.319430 -0.027111 -0.319237
O -0.498688 2.237955 -0.154969
C -1.639359 -0.230876 -0.886877
H 0.232459 -0.827372 -0.036184
C -2.709937 -0.319889 0.193163
H -1.864187 0.607574 -1.555196
H -1.626910 -1.154787 -1.476787
O -3.939658 -0.566258 -0.443303
H -2.468486 -1.135977 0.900703
H -2.742640 0.627709 0.759437
C -5.017015 -0.616457 0.461540
H -5.923498 -0.812053 -0.121076

H -4.881263 -1.425027 1.201406
H -5.131642 0.340349 0.999887

[M-NO2]
E = -642.414375
N -2.586911 -0.785063 -1.069390
N -2.271938 0.013648 -0.028067
C -2.173931 -0.724011 1.095941
N -2.422144 -1.993374 0.836303
C -2.669708 -1.975539 -0.501887
C -2.009911 1.423389 -0.209020
C -0.532175 1.773531 -0.084823
O -0.162409 2.884761 0.244317
N 0.342017 0.785127 -0.493844
C 1.458966 0.481836 0.347029
C 2.566039 -0.198268 -0.439611
O 3.586972 -0.524270 0.470237
C 4.686075 -1.149723 -0.150543
H -1.934072 -0.296739 2.064559
H -2.355074 1.692476 -1.213517
H -2.564168 2.017590 0.525790
H 1.819197 1.399778 0.842392
H 1.115898 -0.194914 1.153337
H 2.171499 -1.103467 -0.934460
H 2.940122 0.483694 -1.224325
H 5.422547 -1.363937 0.630950
H 4.386140 -2.094909 -0.635378
H 5.143660 -0.491573 -0.909407
H -2.921548 -2.861581 -1.073894

[M-NO2]-
E = -642.476651
N -2.382794 0.209629 -0.316731
C -3.588566 0.171324 0.274235
N -4.021564 -1.063061 0.396584
C -3.000370 -1.895841 -0.149197
N -2.003720 -1.099628 -0.591124
H -4.100672 1.081882 0.583978
C -1.534728 1.329437 -0.613664
H -1.379400 1.416330 -1.701685
H -2.016299 2.252439 -0.270811
C -0.140128 1.288853 0.019210
N 0.284087 0.067402 0.392200
O 0.522299 2.322734 0.127804
C 1.602016 -0.148507 0.937455
H -0.337837 -0.717651 0.136320
C 2.632772 -0.326250 -0.169504
H 1.881956 0.713868 1.555011
H 1.579957 -1.044589 1.570106
O 3.898837 -0.558430 0.419675
H 2.349384 -1.178869 -0.813237
H 2.659886 0.585179 -0.792066
C 4.910097 -0.711986 -0.538877
H 5.851088 -0.887642 -0.003494
H 4.709283 -1.571740 -1.204485
H 5.014950 0.193796 -1.163917

[M-NO2]-
E = -642.534494
N -3.609143 0.413824 -0.627141
N -2.361366 0.219551 -0.137510
C -2.257261 -1.026779 0.348956
N -3.407492 -1.682225 0.195822
C -4.191397 -0.752995 -0.403944
C -1.413281 1.336321 -0.179925
C 0.059917 1.128446 0.223528
O 0.744185 2.177019 0.077473
N 0.418891 -0.056584 0.669450
C 1.819695 -0.147892 1.036129
C 2.693625 -0.365722 -0.191139
O 4.047327 -0.580866 0.203192
C 4.896981 -0.723506 -0.896062
H -1.318581 -1.366386 0.773152
H -1.422931 1.717189 -1.207587
H -1.815139 2.130952 0.461974
H 2.179536 0.759442 1.551983
H 1.956249 -1.007106 1.712697
H 2.330732 -1.246207 -0.754047
H 2.628862 0.521174 -0.843804
H 5.915112 -0.886992 -0.517640
H 4.608285 -1.587319 -1.526670
H 4.893361 0.180326 -1.534683
H -5.223494 -0.933496 -0.690305

[M-NO2]-
E = -642.551287
C 5.266166 -0.986117 -0.568633
O 4.235386 -0.901501 0.378272
C 3.012624 -0.480041 -0.195576
C 1.969413 -0.361375 0.907955
N 0.688622 0.033749 0.380392
C 0.361780 1.322840 0.133761
O 1.107324 2.272770 0.373321
C -1.027052 1.494494 -0.478220
N -1.688204 0.236508 -0.706809
C -2.910035 0.134479 -0.277579
N -3.648519 -0.981003 -0.400761
C -4.870702 -0.944043 0.084366
N -5.974260 -1.000164 0.489053
H -3.380977 1.011583 0.219726
H -0.860650 2.041751 -1.422999
H -1.577861 2.185900 0.190233
H -0.013320 -0.634366 0.066939
H 3.133620 0.500892 -0.686928
H 2.301892 0.391045 1.634243
H 1.864653 -1.324437 1.423916
H 2.677153 -1.209702 -0.955945
H 5.024627 -1.713771 -1.365436
H 5.461595 -0.006212 -1.040864
H 6.170957 -1.317887 -0.045192

[M-OH]-
E = -771.812508
C 3.708983 -0.334065 -0.114498
N 2.757201 -0.825710 0.679138
N 1.760369 0.036762 0.500870
C 2.138911 1.006593 -0.371337
N 3.375503 0.796701 -0.785588
C 0.501003 -0.116696 1.240136
C -0.693573 0.566699 0.554191
N -1.749014 -0.206001 0.492767
C -2.918755 0.441982 -0.061658

C -4.068745 -0.546792 -0.027299
O -5.243712 0.046378 -0.567087
C -6.329103 -0.832962 -0.559956
N 4.932804 -1.043286 -0.157696
O -0.542333 1.768070 0.186116
O 5.776498 -0.560787 -0.885275
H 1.450764 1.804035 -0.632846
H 0.325819 -1.188993 1.348547
H 0.648112 0.333344 2.232629
H -3.200028 1.350234 0.502775
H -2.756371 0.773181 -1.103701
H -3.803935 -1.448572 -0.610084
H -4.257050 -0.865776 1.014867
H -7.192074 -0.306966 -0.988828
H -6.125561 -1.738074 -1.164299
H -6.583669 -1.159508 0.467006

M-
E = -847.594653
N -1.482651 1.357931 -0.337219
C -2.632661 1.702918 0.273595
N -3.412706 0.665919 0.458825
C -2.668292 -0.370472 -0.062837
N -1.476720 0.008809 -0.578984
H -2.854717 2.728649 0.556790
N -3.067766 -1.687109 -0.048475
O -2.244601 -2.554465 -0.501692
O -4.217541 -1.981002 0.414033
C -0.344571 2.163842 -0.685963
H -0.184902 2.134022 -1.775134
H -0.548445 3.200870 -0.399454
C 0.983388 1.765548 -0.030881
N 1.066059 0.493023 0.400527
O 1.892105 2.591145 0.040315
C 2.283486 -0.047241 0.961605
H 0.272951 -0.118764 0.175048
C 3.187623 -0.605736 -0.128996
H 2.810371 0.749143 1.499997
H 2.022174 -0.842917 1.669585
O 4.352054 -1.129036 0.477846
H 2.656390 -1.397440 -0.687678
H 3.451017 0.201163 -0.835837
C 5.237996 -1.681728 -0.459520
H 6.106884 -2.061436 0.091007
H 4.770064 -2.515355 -1.013704
H 5.576748 -0.923867 -1.189371

TS, direct dissociation
E = -847.552377
C 0.450579 2.526872 0.787701
N 2.039951 1.617454 0.345875
C 2.291378 1.422824 -0.987881
N 2.533729 0.161338 -1.292981
C 2.372977 -0.430654 -0.087551
N 2.079163 0.395876 0.927296
N 2.462344 -1.833707 0.094893
O 2.961478 -2.519749 -0.801550
O 2.039958 -2.302987 1.159440
C -0.638520 1.776867 0.167894
O -1.271736 2.174235 -0.821829
N -0.864469 0.531750 0.725699
C -1.658278 -0.440723 -0.001194
C -3.138055 -0.252546 0.295951
O -3.876022 -1.233989 -0.410075
C -5.257305 -1.082557 -0.240425
H 2.274890 2.240200 -1.702062
H 0.605001 3.523911 0.378272
H 0.582177 2.431308 1.864821
H -0.075972 0.162627 1.250370
H -1.495595 -0.339569 -1.083401
H -1.342071 -1.444215 0.308085
H -3.318302 -0.352279 1.382790
H -3.445254 0.759405 -0.018515
H -5.605373 -0.100225 -0.609840
H -5.752264 -1.875849 -0.814116
H -5.548335 -1.174874 0.823272

LM upon direct dissociation
E = -847.589190
C 0.379529 3.299503 -0.320107
N -2.650953 1.561882 -0.272685
C -3.831924 0.939166 -0.436145
N -3.786713 -0.391437 -0.278900
C -2.483816 -0.535736 -0.005843
N -1.768872 0.588789 0.010586
N -1.865487 -1.810906 0.252243
O -2.565701 -2.812426 0.232057
O -0.658354 -1.827562 0.479312
C 1.459312 2.414753 0.105624
O 2.643012 2.790251 0.134921
N 1.064665 1.166074 0.471967
C 2.013796 0.161599 0.886021
C 2.509363 -0.647732 -0.304779
O 3.459348 -1.594291 0.152081
C 3.892024 -2.445222 -0.873041
H -4.742644 1.481546 -0.676681
H 0.653222 4.312361 -0.609456
H -0.661824 2.971749 -0.356220
H 0.078039 0.900508 0.346862
H 2.865436 0.655271 1.369668
H 1.525306 -0.511498 1.600423
H 1.655376 -1.162327 -0.778276
H 2.972582 0.030299 -1.044060
H 4.374207 -1.879531 -1.692469
H 4.623550 -3.140120 -0.442005
H 3.052137 -3.024836 -1.297610

TS H transfer to ring N atom
E = -847.557372
n -1.311318 -0.719499 0.635045
h 1.687209 0.022586 -1.598352
n 2.044012 -0.435569 -0.766409
c 2.776377 0.242728 0.176693
n 2.544392 -0.238223 1.417187
c 1.596867 -1.144582 1.226078
n 1.255014 -1.301660 -0.052782
c 0.190227 -2.080379 -0.660939
c -1.245405 -1.608997 -0.324222
o -2.129324 -2.188340 -1.005731
n 3.604657 1.253043 -0.220263
o 3.557812 1.536292 -1.463506
o 4.332543 1.837597 0.618996

c -2.659194 -0.301320 0.971889
c -3.137367 0.788877 0.023435
o -4.413082 1.265084 0.437150
c -4.913094 2.245510 -0.425238
h -5.891472 2.563625 -0.041916
h -4.244333 3.126403 -0.472555
h -5.040343 1.856733 -1.453313
h -2.411986 1.623530 0.022141
h -3.202988 0.379821 -0.999795
h -2.662468 0.105685 1.994863
h -3.381146 -1.135116 0.927040
h 0.298915 -3.126079 -0.345189
h 1.118856 -1.729942 2.002135
h 0.338403 -2.049978 -1.745946

LM H transfer to ring N atom
E = -847.561184
h 2.717868 1.367244 1.005261
o 4.922363 0.509745 0.945313
c 4.454070 -0.489561 0.300496
o 5.107230 -1.521568 0.010009
c 3.141109 -0.382203 -0.061086
n 2.463177 0.772930 0.224342
n 1.148931 0.512422 -0.085516
c 1.143149 -0.697349 -0.664195
n 2.336595 -1.281738 -0.664244
c 0.168398 1.595019 0.010847
c -1.316734 1.277591 -0.273857
o -2.062499 2.258926 -0.033060
n -1.588887 0.078595 -0.735922
c -2.998345 -0.149111 -1.000863
c -3.742473 -0.513410 0.276269
o -5.090421 -0.850031 -0.030695
c -5.834963 -1.140067 1.116743
h -5.411469 -1.999898 1.671020
h -6.856004 -1.390731 0.800790
h -5.874928 -0.275052 1.805255
h -3.716658 0.343568 0.970954
h -3.247106 -1.374708 0.762688
h -3.096440 -0.986984 -1.708414
h -3.488603 0.735139 -1.441953
h 0.479078 2.392837 -0.678042
h 0.198347 -1.070514 -1.050976
h 0.224484 1.991161 1.033633

TS H transfer from ring to NO2
E = -847.549782
h -3.376922 1.272365 -0.568045
o -4.738277 0.819877 -0.680736
c -4.455241 -0.413774 -0.253992
o -5.299215 -1.328212 -0.134363
c -3.110106 -0.521539 0.027857
n -2.488840 0.634120 -0.206451
n -1.185790 0.412398 0.095109
c -1.108964 -0.869024 0.496417
n -2.293373 -1.491772 0.468842
c -0.204966 1.493128 -0.046301
c 1.276238 1.210560 0.284514
o 1.996375 2.224828 0.109193
n 1.580179 0.000791 0.697837
c 2.988607 -0.185946 0.997585
c 3.787191 -0.452706 -0.270722
o 5.137712 -0.752275 0.063510
c 5.929961 -0.947521 -1.071776
h 5.560977 -1.791411 -1.686435
h 6.950112 -1.174238 -0.735332
h 5.955488 -0.044550 -1.710444
h 3.747998 0.438512 -0.920015
h 3.343403 -1.305897 -0.817632
h 3.098361 -1.055372 1.664314
h 3.428686 0.692335 1.499438
h -0.536924 2.317677 0.597255
h -0.135169 -1.254555 0.788126
h -0.255065 1.837329 -1.086908

LM H transfer from ring to NO2
E = -847.582036
h 4.286133 1.311442 0.751160
o 5.034278 0.687926 0.695234
c 4.444672 -0.481803 0.222597
o 5.178023 -1.462232 0.051130
c 3.074377 -0.396254 -0.004271
n 2.460323 0.756533 0.241821
n 1.179216 0.464686 -0.085266
c 1.104709 -0.811206 -0.497528
n 2.301261 -1.399149 -0.459728
c 0.165650 1.516427 0.049039
c -1.309543 1.212303 -0.282848
o -2.048077 2.215214 -0.103723
n -1.606102 0.001662 -0.702746
c -3.013502 -0.192129 -1.000216
c -3.808396 -0.465594 0.268955
o -5.159392 -0.774279 -0.060833
c -5.945185 -0.975188 1.077143
h -5.568333 -1.817067 1.690206
h -6.965707 -1.208340 0.745505
h -5.973806 -0.072961 1.717036
h -3.773421 0.425565 0.918370
h -3.358151 -1.316726 0.813754
h -3.120180 -1.061509 -1.668194
h -3.460627 0.684612 -1.499304
h 0.480904 2.350249 -0.590281
h 0.143773 -1.217964 -0.798776
h 0.206516 1.866014 1.087655
TS H transfer to ring C atom
E = -847.521997
n -2.174665 1.941671 0.189769
c -1.825269 0.545712 -0.057719
h -0.622909 0.357456 -0.318177
n 0.549377 -0.698723 -0.444662
n -1.822683 -0.310655 1.078941
n -1.883394 -1.532649 0.446811
c -2.312256 -1.324293 -0.870169
n -2.454922 -0.093734 -1.189338
h -2.527954 -2.173633 -1.515678
c -0.749742 -2.416121 0.710484
c 0.590089 -1.891954 0.144574
c 1.798945 -0.224238 -1.001862
c 2.649354 0.446033 0.068435
o 3.826500 0.980785 -0.523127
c 4.659821 1.589322 0.421402
h 4.150881 2.428666 0.932280

h 1.577434 0.513564 -1.787940
h 2.071845 1.256451 0.550189
h 2.916704 -0.296882 0.839332
h 2.380937 -1.045659 -1.448708
o 1.569604 -2.655068 0.278477
h -0.950700 -3.399622 0.264285
h -0.654999 -2.541520 1.794765
h 4.993176 0.869528 1.192583
h 5.539949 1.976905 -0.107911
o -1.949346 2.388079 1.307222
o -2.601259 2.607029 -0.744639

LM H transfer to ring C atom
E = -847.549534
n -4.297969 -0.444078 -0.165143
c -2.795486 -0.922699 -0.296532
h -2.910914 -1.856864 -0.859343
n 1.446378 0.262115 0.662308
n -2.072178 0.086200 -1.017052
n -1.197636 0.519849 -0.110896
c -1.315553 -0.188964 1.062948
n -2.251952 -1.079411 1.022162
h -0.634222 0.009038 1.881628
c -0.266920 1.596810 -0.411519
c 1.209026 1.357675 -0.024956
c 2.840020 0.069688 1.015280
c 3.617988 -0.531145 -0.147019
o 4.950984 -0.826651 0.260586
c 5.722731 -1.327241 -0.791221
h 5.298661 -2.265905 -1.198245
h 2.901313 -0.626616 1.866882
h 3.121014 -1.459314 -0.485867
h 3.631359 0.186188 -0.985245
h 3.334263 1.013938 1.303781
o 1.980630 2.272780 -0.410020
h -0.608621 2.513694 0.089923
h -0.329812 1.762624 -1.490847
h 5.800801 -0.600032 -1.621551
h 6.729234 -1.532964 -0.403147
o -4.512703 0.576936 0.449488
o -5.142714 -1.133220 -0.709994

TS direct NO2 dissociation
E = -847.549914
n -1.370874 2.384968 -0.131056
c -2.542254 0.891816 0.422951
n -1.846558 -0.020630 1.090853
n -2.131462 -1.197580 0.431235
c -2.956579 -0.916265 -0.599005
n -3.257923 0.363149 -0.638751
o -0.177984 2.037283 -0.142690
o -1.697406 3.555726 -0.004255
c -1.245956 -2.319802 0.589055
c 0.158794 -2.143921 -0.005743
n 0.508791 -0.897799 -0.370536
o 0.874772 -3.139546 -0.119960
c 1.820528 -0.581195 -0.891183
c 2.659964 0.102393 0.178041
o 3.927886 0.409921 -0.373162
c 4.722259 1.150719 0.513705
h 5.679406 1.348097 0.015388
h 4.245108 2.112088 0.774779
h 4.913115 0.591523 1.448803
h 2.775654 -0.571237 1.047119
h 2.148529 1.021409 0.511207
h 2.299805 -1.510234 -1.217869
h 1.710176 0.094594 -1.748640
h -0.080550 -0.099494 -0.119449
h -1.687097 -3.203372 0.113000
h -1.124793 -2.533471 1.658933
h -3.311858 -1.684202 -1.281873

LM direct NO2 dissociation
E = -847.551356
n -1.108184 2.522211 -0.020126
c -2.691281 0.942309 0.525093
n -1.995800 0.010319 1.150344
n -2.213094 -1.105188 0.378843
c -3.004071 -0.767486 -0.656978
n -3.344745 0.506844 -0.597631
o -0.106895 1.895422 -0.417918
o -1.071972 3.741673 0.055409
c -1.376135 -2.262955 0.538855
c 0.054701 -2.132633 0.002426
n 0.439211 -0.921502 -0.431888
o 0.757632 -3.144256 -0.012407
c 1.783289 -0.672119 -0.909419
c 2.615335 -0.004788 0.175754
o 3.919196 0.217684 -0.331345
c 4.713521 0.950303 0.562301
h 5.699787 1.079651 0.099623
h 4.276885 1.943863 0.767591
h 4.835580 0.418033 1.524304
h 2.659579 -0.659238 1.065734
h 2.141210 0.949587 0.460525
h 2.234401 -1.626300 -1.202081
h 1.728792 -0.008103 -1.780752
h -0.130689 -0.081418 -0.282105
h -1.834692 -3.116219 0.024887
h -1.305737 -2.505767 1.605906
h -3.302678 -1.482838 -1.418526

TS ring opening
E = -847.539011
n -1.469694 -0.398346 0.186220
n -1.069287 1.309694 -0.408416
c -2.257679 1.640709 -0.898107
n -3.200451 0.717841 -0.953116
c -2.689875 -0.340539 -0.245661
n -3.657079 -1.338381 0.212798
o -4.629723 -1.572012 -0.509665
o -3.400693 -1.982323 1.236301
c -0.142711 2.276554 0.127412
c 1.221811 1.678433 0.457252
n 1.245442 0.337213 0.589127
o 2.204164 2.403700 0.622464
c 2.456869 -0.369434 0.930003
c 3.277646 -0.681491 -0.314061
o 4.439023 -1.391170 0.073496
c 5.263792 -1.696546 -1.018260
h 5.610584 -0.780626 -1.530889
h 6.133990 -2.242552 -0.634228

h 4.738975 -2.330330 -1.756737
h 2.677523 -1.286300 -1.018226
h 3.553092 0.262941 -0.815298
h 2.187067 -1.302808 1.439158
h 3.057121 0.246433 1.611244
h -0.506954 2.742761 1.060809
h -2.481920 2.668566 -1.205349
h 0.369195 -0.174616 0.445747
h 0.037518 3.088708 -0.594766

LM ring opening
E = -847.567868
n -1.799629 -0.650700 0.935644
n -0.858465 1.624146 -0.005097
c -1.873216 1.607069 -0.803979
n -2.882986 0.711483 -0.771575
c -2.692675 -0.291981 0.101476
n -3.869183 -1.275146 0.042247
o -3.776021 -2.217793 -0.722640
o -4.818427 -1.057129 0.771972
c 0.208768 2.562218 -0.218605
c 1.562966 1.926909 0.098335
n 1.469529 0.711094 0.678235
o 2.626870 2.500823 -0.137857
c 2.624651 -0.078338 1.022782
c 3.060802 -0.949407 -0.147721
o 4.178873 -1.720846 0.249680
c 4.646467 -2.547614 -0.781812
h 4.967596 -1.956241 -1.658853
h 5.506197 -3.107604 -0.394224
h 3.869852 -3.262380 -1.110396
h 2.229463 -1.610229 -0.453649
h 3.323625 -0.303866 -1.004339
h 2.380885 -0.713040 1.884047
h 3.445422 0.595215 1.298605
h 0.126057 3.431113 0.458239
h -1.974007 2.361097 -1.605676
h 0.520167 0.345487 0.765974
h 0.272948 2.960737 -1.247998

TS NO2 dissociation upon ring opening
E = -847.560161
n -4.295922 -1.173185 -0.026064
c -2.707706 -0.181537 0.227511
n -2.752404 0.701522 -0.753193
c -1.690471 1.542539 -0.803493
n -0.717356 1.607363 0.042186
c 0.364081 2.517277 -0.229091
c 1.720939 1.878890 0.064104
n 1.635153 0.681814 0.684859
o 2.783104 2.430453 -0.223241
c 2.792461 -0.107144 1.024845
c 3.212829 -0.996639 -0.137669
o 4.334842 -1.761615 0.258805
c 4.795337 -2.599984 -0.767021
o -5.269228 -0.802077 0.586100
o -4.224382 -2.120539 -0.772618
h -1.716931 2.213809 -1.680741
h 0.403909 2.886939 -1.270789
h 0.311466 3.406369 0.423785
h 0.684296 0.342277 0.821575
n -2.213366 -0.740538 1.195557
h 3.464706 -0.364535 -1.007321
h 3.618930 0.567750 1.279912
h 2.559225 -0.727349 1.899460
h 2.377873 -1.662287 -0.423329
h 4.017139 -3.319741 -1.080474
h 5.108228 -2.018321 -1.653295
h 5.658928 -3.153951 -0.379681

LM NO2 dissociation upon ring opening
E = -847.594237
n -1.397024 -1.705870 -0.949175
c -3.479968 -0.726024 1.184444
n -3.779636 0.531444 0.919410
c -2.872802 1.264769 0.256736
n -1.709902 0.885710 -0.185433
c -0.904985 1.865133 -0.865884
c 0.547138 1.862130 -0.391681
n 0.806420 0.949318 0.570194
o 1.392295 2.624062 -0.861316
c 2.134273 0.697342 1.071479
c 2.872176 -0.306652 0.195844
o 4.155882 -0.531486 0.749168
c 4.905639 -1.443602 -0.007452
o -1.934586 -1.814092 -2.008042
o -0.307125 -2.014457 -0.570415
h -3.200931 2.307733 0.092388
h -0.854389 1.662023 -1.950219
h -1.271005 2.904590 -0.758754
h 0.022500 0.341334 0.795465
n -3.309354 -1.852060 1.473384
h 2.959922 0.098626 -0.827393
h 2.689759 1.642973 1.086837
h 2.064306 0.312599 2.096848
h 2.304383 -1.252320 0.146188
h 4.405878 -2.427805 -0.065091
h 5.067930 -1.076061 -1.037258
h 5.877570 -1.564285 0.486254

M(H2O)
E = -923.954173
N 1.428992 1.431066 -0.367732
C 2.273033 1.875678 0.588439
N 3.187691 0.961446 0.859229
C 2.819876 -0.030291 0.032912
N 1.770676 0.189792 -0.734258
H 2.185152 2.854687 1.046436
N 3.517661 -1.307336 -0.031677
O 2.909704 -2.244991 -0.533900
O 4.640731 -1.353077 0.413754
C 0.246366 2.077958 -0.907930
H 0.166386 1.798412 -1.964238
H 0.380403 3.160828 -0.836180
C -1.067281 1.737862 -0.187738
N -1.196765 0.481551 0.277781
O -1.915084 2.613743 -0.090422
C -2.423594 0.035132 0.912350
H -0.527553 -0.247034 0.038332
C -3.435600 -0.452093 -0.115994
H -2.847783 0.866208 1.485942
H -2.174811 -0.780473 1.600984

O -4.556960 -0.939305 0.581673
H -2.984552 -1.249486 -0.735200
H -3.720333 0.383523 -0.780013
C -5.578893 -1.379633 -0.279787
H -6.401465 -1.741098 0.346634
H -5.229526 -2.202391 -0.928466
H -5.945295 -0.557444 -0.919011
O -0.025418 -2.151667 -0.177316
H -0.182749 -2.977291 0.285121
H 0.897107 -2.189380 -0.467576

M(H2O)2
E = -1000.372145
N -1.024735 -1.655984 -0.421639
C -1.948219 -2.006494 0.499169
N -2.857853 -1.058300 0.626660
C -2.406712 -0.143936 -0.249679
N -1.309522 -0.442418 -0.913838
H -1.917243 -2.945651 1.040302
N -3.084231 1.126870 -0.477344
O -2.440793 2.007003 -1.029907
O -4.233308 1.215220 -0.105557
C 0.129188 -2.397095 -0.895105
H 0.137486 -2.366798 -1.988741
H 0.013478 -3.440218 -0.580051
C 1.496953 -1.902589 -0.415177
N 1.522031 -1.003820 0.581382
O 2.489342 -2.369836 -0.959449
C 2.782485 -0.479949 1.075946
H 0.669549 -0.687783 1.044648
C 3.456109 0.480869 0.111227
H 3.476002 -1.310742 1.262779
H 2.573698 0.025751 2.025413
O 2.673430 1.654136 -0.023635
H 3.591835 -0.000894 -0.869669
H 4.451193 0.745445 0.509568
C 3.224358 2.577495 -0.940717
H 2.555581 3.444339 -0.970482
H 3.299074 2.136456 -1.947986
H 4.225600 2.903301 -0.614809
O 0.033935 2.305216 0.575276
H 0.920593 2.019764 0.292457
H -0.536583 2.224880 -0.199382
O -0.391106 0.245103 2.259722
H -0.361562 1.091369 1.760216
H -1.275500 0.171787 2.623618

M(H2O)3
E = -1076.783890
N 1.323904 -1.364623 -0.411897
C 2.410159 -1.774496 -1.093974
N 3.412482 -0.929677 -0.923291
C 2.847184 -0.004773 -0.132829
N 1.589897 -0.206465 0.205452
H 2.425475 -2.679472 -1.690241
N 3.554807 1.178676 0.347993
O 2.906664 1.970460 1.009291
O 4.726506 1.279933 0.051306
C -0.018031 -1.909651 -0.394452
H -0.357306 -1.969464 0.645162
H 0.024966 -2.919856 -0.816730
C -0.921518 -1.033842 -1.274489
N -2.165210 -0.850385 -0.801759
O -0.494244 -0.558897 -2.318667
C -3.106690 0.007764 -1.493324
H -2.453733 -1.295935 0.072693
C -2.763095 1.482886 -1.361318
H -4.099921 -0.183303 -1.071052
H -3.123876 -0.245594 -2.561766
O -2.754571 1.838925 0.011464
H -3.522735 2.077039 -1.898029
H -1.779088 1.685700 -1.813147
C -2.538343 3.225245 0.207164
H -2.505492 3.398648 1.287426
H -3.357174 3.813847 -0.236458
H -1.581766 3.542842 -0.238728
O -0.658142 0.937289 1.712438
H -1.372443 1.114850 1.076740
H 0.153253 0.829637 1.191422
O -2.953985 -2.283440 1.556409
H -3.808672 -2.158553 1.972715
H -2.285413 -2.041464 2.231695
O -0.833743 -1.414539 2.944315
H -0.566060 -1.386124 3.864085
H -0.767041 -0.492113 2.595618

M(H2O)4
E = -1153.197223
N 1.027539 1.622284 0.587883
C 1.598677 2.120632 -0.532192
N 2.639332 1.385508 -0.878971
C 2.630097 0.443393 0.078758
N 1.689638 0.531949 0.994821
H 1.216726 2.994064 -1.046823
N 3.572321 -0.664615 0.110390
O 3.366679 -1.546488 0.932184
O 4.488217 -0.640691 -0.682901
C -0.171816 2.048595 1.288079
H -0.230709 1.442928 2.196094
H -0.083935 3.109771 1.548461
C -1.394703 1.881810 0.382947
N -2.349777 1.079142 0.868139
O -1.440511 2.482371 -0.691949
C -3.544602 0.727038 0.122283
H -2.102185 0.478827 1.659256
C -3.651435 -0.779025 -0.044986
H -4.437054 1.089728 0.651545
H -3.500528 1.227447 -0.851543
O -2.573837 -1.221204 -0.839942
H -3.610698 -1.273655 0.940293
H -4.614706 -1.030096 -0.521945
C -2.394634 -2.626129 -0.831252
H -1.526865 -2.838762 -1.463175
H -2.190625 -2.981284 0.190079
H -3.283312 -3.138808 -1.233470
O 0.813156 -1.387012 -1.575718
H 0.264760 -0.690541 -1.999363
H 1.412565 -1.712168 -2.249542
O -1.026542 0.335550 -2.557996
H -1.092813 1.215907 -2.159732
H -1.703659 -0.177662 -2.085096
O 0.396906 -2.280437 1.012481

H 0.435458 -2.021557 0.071956
H 1.281879 -2.048599 1.323106
O -1.363286 -0.929070 2.539438
H -0.717401 -1.418436 1.971785
H -1.286025 -1.309030 3.415682

M(H2O)5
E = -1229.608804
-0.966382 1.683066 0.283963
-1.414388 2.126000 -0.910610
-2.408682 1.366947 -1.338791
-2.501353 0.466223 -0.351197
-1.666682 0.604820 0.656731
-0.980811 2.980075 -1.417091
-3.429647 -0.653445 -0.365495
-3.363445 -1.434757 0.574527
-4.190247 -0.737892 -1.303014
0.155225 2.147661 1.086631
0.105360 1.614177 2.038919
0.049526 3.224096 1.259432
1.464636 1.916546 0.326405
2.293276 1.031650 0.884971
1.666956 2.544645 -0.716582
3.560827 0.647732 0.290208
1.909555 0.409324 1.614384
3.648196 -0.858293 0.117024
4.387653 0.974881 0.936498
3.661917 1.158843 -0.673890
2.679909 -1.262736 -0.826396
3.464105 -1.364999 1.079489
4.660917 -1.128050 -0.229672
2.537235 -2.668394 -0.927674
1.755919 -2.855447 -1.670778
2.222876 -3.094948 0.036212
3.480622 -3.135387 -1.253995
1.138618 -0.946856 2.449332
0.400170 -0.736538 3.042934
0.704931 -1.478617 1.749336
1.370353 0.411832 -2.616746
1.452707 1.283317 -2.201147
1.972594 -0.147683 -2.096006
-0.331743 -2.384499 0.579776
-0.368002 -2.024254 -0.326860
-1.242828 -2.312006 0.883311
-0.633833 -1.258404 -1.922061
-0.008199 -0.572943 -2.246244
-1.168918 -1.517924 -2.674086
-1.364875 -0.117364 3.424139
-1.658125 -0.040372 2.499727
-2.030959 -0.643428 3.871355

M-(H2O)
E = -924.020174
O -0.239275 -2.091483 -0.226580
H -0.140874 -2.650614 -0.999252
H 0.658584 -2.152976 0.209139
N 1.529962 1.542835 0.357017
C 2.568519 1.816706 -0.454478
N 3.319753 0.755859 -0.647482
C 2.660745 -0.206323 0.078985
N 1.567556 0.233109 0.725307
H 2.737047 2.804986 -0.872887
N 3.039267 -1.532325 0.119791
O 2.233880 -2.362354 0.714333
O 4.131431 -1.897650 -0.410695
C 0.407423 2.363658 0.723676
H 0.272094 2.323179 1.813466
H 0.624557 3.399869 0.443811
C -0.937846 2.001717 0.082375
N -1.073383 0.749269 -0.384625
O -1.814922 2.864864 0.045604
C -2.324418 0.270665 -0.931912
H -0.357888 0.044416 -0.191540
C -3.141428 -0.437195 0.138636
H -2.882969 1.123673 -1.332576
H -2.102409 -0.433840 -1.742009
O -4.341303 -0.906292 -0.450972
H -2.551863 -1.276820 0.542923
H -3.371055 0.269476 0.957033
C -5.118681 -1.644689 0.452660
H -6.020898 -1.973524 -0.077555
H -4.573314 -2.531278 0.822903
H -5.417472 -1.031680 1.323314

M-(H2O)2
E = -1000.440608
O -0.130687 1.306160 -2.456836
H 0.447608 1.986999 -2.069329
H 0.308594 0.487488 -2.150630
N -0.491716 -1.798664 -0.613251
C -0.166516 -2.440098 0.524994
N 1.053816 -2.144360 0.913421
C 1.451393 -1.236973 -0.034810
N 0.543909 -0.998581 -0.989279
H -0.856514 -3.108266 1.030166
N 2.661986 -0.574559 -0.003900
O 2.878141 0.307549 -0.939890
O 3.501682 -0.822808 0.910960
C -1.803727 -1.611163 -1.199120
H -1.655093 -1.274864 -2.231666
H -2.344904 -2.562557 -1.186179
C -2.594855 -0.597264 -0.362176
N -2.233564 0.686997 -0.555961
O -3.438918 -0.986228 0.440647
C -2.451217 1.698043 0.458631
H -1.488853 0.890690 -1.234546
C -1.140398 2.040119 1.155075
H -2.863826 2.609946 0.002244
H -3.179581 1.303747 1.176114
O -0.664042 0.887135 1.803350
H -0.398317 2.397752 0.420035
H -1.316327 2.856332 1.884536
C 0.666063 1.026591 2.261804
H 0.997797 0.043226 2.609524
H 1.332901 1.361537 1.453194
H 0.721265 1.754896 3.092137
O 1.747349 2.657596 -0.873072
H 2.177427 1.749139 -0.825049
H 2.464168 3.237327 -1.135421

M-(H2O)3
E = -1076.860462

1.544531 -1.837687 0.313865
1.370999 -2.057642 -1.008568
0.098721 -2.053851 -1.333516
-0.502849 -1.756403 -0.135179
0.332726 -1.629885 0.896547
2.203974 -2.221386 -1.685475
-1.855950 -1.520917 0.007558
-2.281860 -1.213562 1.197531
-2.620410 -1.521777 -1.016414
2.712315 -1.270516 0.959127
2.567832 -1.371547 2.040471
3.612025 -1.807687 0.643263
2.862169 0.195516 0.527065
1.837973 0.998419 0.861964
3.837001 0.548706 -0.138967
1.637835 2.267136 0.192713
1.017750 0.645216 1.373781
1.099954 2.069891 -1.219564
0.919642 2.843551 0.787490
2.586006 2.816824 0.140724
-0.126615 1.380658 -1.135391
0.962828 3.052358 -1.709545
1.821077 1.483457 -1.813927
-0.637504 0.951593 -2.382331
-1.509647 0.321066 -2.173085
-0.923363 1.816921 -3.006422
0.108607 0.342108 -2.915344
-2.101635 2.580027 0.530205
-1.466844 2.200367 -0.097537
-2.879672 1.989900 0.448746
-0.638135 0.864452 2.321791
-1.177106 1.544058 1.881517
-1.080348 0.034634 2.056679
-4.087342 0.619313 0.346593
-3.557458 -0.023239 0.873155
-4.016858 0.180854 -0.511783

M-(H2O)4
E = -1153.273956
7 0.373267 -1.994271 -0.521255
6 0.147412 -1.334955 -1.671556
7 -1.087063 -0.876235 -1.712726
6 -1.587517 -1.259730 -0.493843
7 -0.733267 -1.946617 0.268833
1 0.921807 -1.185715 -2.417909
7 -2.850492 -0.909737 -0.046051
8 -3.093478 -1.001314 1.218105
8 -3.703863 -0.410073 -0.867630
6 1.644423 -2.379022 0.042220
1 1.457564 -3.144917 0.803779
1 2.280576 -2.798083 -0.743187
6 2.411181 -1.196298 0.645677
7 1.670512 -0.288869 1.291897
8 3.633534 -1.122308 0.479415
6 2.178044 1.007144 1.685347
1 0.677141 -0.444090 1.503215
6 1.437080 2.119907 0.961691
1 2.037361 1.145857 2.766268
1 3.249498 1.040386 1.459006
8 1.718708 2.057938 -0.423276
1 0.351862 2.021371 1.124855
1 1.758183 3.097516 1.366102
6 0.903649 2.930867 -1.181076
1 1.155284 2.773465 -2.235747
1 -0.164872 2.719243 -1.024699
1 1.105605 3.983027 -0.915293
8 -2.361351 1.794636 -1.645284
1 -1.671284 1.199482 -1.970210
1 -3.058741 1.122899 -1.463118
8 3.079562 0.121466 -2.098659
1 3.594085 -0.360004 -1.437181
1 2.616574 0.779396 -1.556511
8 -0.844796 0.062701 2.579002
1 -1.242032 0.886692 2.248843
1 -1.495777 -0.585400 2.245361
8 -2.501435 1.922699 1.230636
1 -3.047859 1.122024 1.301835
1 -2.356360 2.003512 0.270470

M-(H2O)5
E = -1229.688968
0.238239 -2.310972 -0.060858
-0.476947 -2.403242 -1.192703
-1.681648 -1.896418 -1.037151
-1.647521 -1.458427 0.257920
-0.504185 -1.708461 0.903885
-0.080133 -2.845717 -2.100533
-2.682194 -0.753609 0.840686
-2.475727 -0.215866 1.995685
-3.771873 -0.588447 0.183454
1.605824 -2.706029 0.174476
1.692309 -2.967949 1.236506
1.827362 -3.593939 -0.425948
2.687312 -1.673240 -0.176457
2.499945 -0.418985 0.259387
3.683987 -2.045377 -0.796099
3.529275 0.585337 0.076772
1.726455 -0.188341 0.892784
2.994354 1.983362 0.313561
4.362649 0.407405 0.776026
3.936715 0.501229 -0.938401
2.223738 2.402910 -0.797469
2.382707 2.003711 1.230592
3.844146 2.676374 0.451553
1.498972 3.587994 -0.526087
0.936717 3.837811 -1.432171
0.791528 3.439046 0.304820
2.184614 4.418399 -0.282613
-0.123513 0.832834 -0.982480
-0.780932 0.821683 -1.710310
0.700464 1.231346 -1.283378
0.756152 0.402413 2.433148
0.192777 1.158323 2.192101
0.152236 -0.344321 2.274767
-1.221393 2.072943 1.218585
-0.841303 1.659754 0.414900
-1.756523 1.341947 1.608867
-2.327469 0.610429 -2.558880
-2.485291 -0.277151 -2.198943
-2.857991 1.188906 -1.972835
-3.661283 2.046875 -0.545748
-2.946398 2.345660 0.035340

-3.881767 1.160693 -0.181226

(NRT-yl)-(H2O)
E = -522.505140
O 2.878131 -1.485178 -0.000315
H 3.097437 -0.549590 -0.000226
H 1.899087 -1.439380 -0.000128
N -2.203831 -1.110058 -0.000083
C -1.047636 -1.798141 0.000130
N 0.049171 -1.030404 0.000194
C -0.527722 0.185013 0.000056
N -1.860244 0.190889 -0.000096
H -1.014878 -2.884860 0.000182
N 0.239413 1.401707 0.000062
O -0.356626 2.470272 -0.000353
O 1.465863 1.313364 0.000483

(NRT-yl)-(H2O)2
E = -598.921357
7 0.976734 1.270544 -0.084553
6 0.004466 2.195413 -0.025613
7 -1.236448 1.690350 0.020696
6 -0.946197 0.383755 -0.011194
7 0.349709 0.082425 -0.075100
1 0.219059 3.260552 -0.015121
7 -1.952096 -0.650078 0.032486
8 -1.574493 -1.814104 0.104153
8 -3.127818 -0.314716 -0.000850
8 3.753801 0.301899 0.157670
1 2.929017 0.812019 0.072831
1 3.434272 -0.608662 0.080213
8 2.065182 -2.123340 -0.197632
1 1.384664 -1.408949 -0.209414
1 1.784694 -2.680566 0.530913

(NRT-yl)-(H2O)3
E = -675.335771
7 0.528934 1.454249 -0.074964
6 -0.535901 2.265130 0.004180
7 -1.712883 1.624054 0.045973
6 -1.282253 0.359671 -0.011614
7 0.038093 0.198442 -0.084964
1 -0.439999 3.346917 0.032491
7 -2.174767 -0.779068 0.006070
8 -1.673205 -1.895356 0.011918
8 -3.378152 -0.565717 0.015264
8 2.096514 -1.538704 -1.337540
1 1.277049 -1.066505 -1.103446
1 2.357156 -1.889345 -0.475182
8 3.313388 0.907008 -0.198870
1 3.168144 0.183030 -0.827039
1 2.420654 1.308467 -0.148489
8 2.289608 -1.132674 1.605438
1 1.387574 -0.914090 1.331472
1 2.797488 -0.397480 1.220325

(NRT-yl)-(H2O)4
E = -751.751470
7 -0.053410 1.852543 -0.046487
6 1.168763 2.405012 -0.018711
7 2.175203 1.517990 0.003885
6 1.472727 0.381863 -0.011256
7 0.148014 0.519193 -0.041420
1 1.318542 3.480852 -0.014960
7 2.083990 -0.926819 0.004840
8 1.346543 -1.909448 -0.008877
8 3.300681 -0.994361 0.031138
8 -2.979491 1.601460 0.072127
1 -2.072527 1.951993 0.002466
1 -2.886477 0.935298 0.775892
8 -1.702833 -2.597093 -0.061307
1 -0.747912 -2.455151 -0.015332
1 -1.982087 -1.953536 -0.741362
8 -1.989477 -0.462871 1.819176
1 -1.176884 -0.137874 1.399560
1 -2.106838 -1.309551 1.354158
8 -2.079427 -0.394809 -1.767429
1 -2.633376 0.257530 -1.301098
1 -1.205940 -0.174191 -1.403888

(NRT-yl)-(H2O)5
E = -828.165929
N 0.304269 1.522797 0.284236
C 1.420451 2.261074 0.164800
N 2.539773 1.542077 0.007513
C 2.022856 0.309269 0.040690
N 0.703498 0.242057 0.201472
H 1.401288 3.346407 0.196507
N 2.819784 -0.889661 -0.079452
O 2.243750 -1.970090 0.008576
O 4.019783 -0.769125 -0.259627
O -0.875719 -1.993486 0.417771
H -0.321710 -1.175942 0.332010
H -0.220334 -2.694574 0.426949
O -2.502506 1.818650 0.895625
H -1.527844 1.773793 0.834844
H -2.772446 0.947748 1.241412
O -2.311884 1.197381 -1.793811
H -2.546777 1.508362 -0.892683
H -1.350306 1.168927 -1.746381
O -3.134965 -1.475049 -1.287767
H -2.308242 -1.807335 -0.901369
H -2.895804 -0.580180 -1.593991
O -3.276810 -0.867352 1.503777
H -2.422608 -1.325537 1.510677
H -3.559517 -1.032047 0.586344

NO2-(H2O)
E = -281.537851
N -1.290784 0.043423 -0.000000
O -0.561807 1.067238 0.000003
O -0.701638 -1.059692 -0.000004
H 1.292439 0.609572 -0.000016
O 2.043190 -0.018859 -0.000005
H 1.505081 -0.823032 0.000060

NO2-(H2O)2
E = -357.955703
N -0.189368 0.876199 0.000306
O 0.972365 1.324407 -0.000196
O -0.297705 -0.379108 0.000193
H 1.848295 -1.464604 0.000033

O 2.665807 -0.953784 -0.000068
H 2.267602 -0.059934 -0.000315
H -3.175755 0.406016 -0.000395
O -3.036016 -0.543583 -0.000180
H -2.050179 -0.598330 0.000547

NO2-(H2O)3
E = -434.371232
N 0.206981 -0.781532 -0.002270
O 0.396463 0.468620 -0.115370
O 1.217531 -1.482626 0.075593
H 3.188465 -0.231161 0.072840
O 3.246055 0.729270 0.003861
H 2.292265 0.917428 -0.068159
H -2.044773 1.926180 0.911684
O -1.985670 1.747440 -0.029218
H -1.102894 1.304241 -0.119858
H -2.797308 -0.130927 -0.105340
O -2.771004 -1.095277 -0.019604
H -1.811617 -1.254455 0.002627

NO2-(H2O)4
E = -510.787968
O -0.055336 2.099787 -0.026243
O 1.489179 0.060769 -1.096616
N 2.226231 -0.276669 -0.128429
O 1.722732 -0.149019 1.004542
O -0.933899 -1.517922 -1.254536
O -2.495840 0.688516 -0.180853
H 0.436854 1.411474 -0.528548
H 0.241458 1.899110 0.867679
H -0.106000 -1.014788 -1.347695
H -0.942584 -1.711791 -0.302874
H -2.155578 -0.012760 -0.761985
H -1.755867 1.323963 -0.159521
O -0.939079 -1.044497 1.610054
H -1.550746 -0.384121 1.245561
H -0.053222 -0.675473 1.435605

NO2-(H2O)5
E = -587.205225
O 2.586253 -0.544855 -0.056492
O 0.916491 0.595380 -2.060394
O -0.767852 -1.559121 -0.922806
N -0.167628 -1.567038 0.167408
O -0.830503 -1.198470 1.170638
O -2.667701 0.516650 -0.011340
O -0.342759 2.209030 -0.135850
H 1.886635 -1.203957 0.087713
H 2.228270 -0.058369 -0.823471
H 0.368932 -0.195800 -1.942599
H 0.486190 1.247927 -1.469662
H -1.202992 1.754693 -0.078732
H 0.148839 1.871723 0.636841
H -2.528429 0.073467 -0.856161
H -2.180706 -0.104556 0.571197
O 1.077887 0.851259 1.941569
H 0.425411 0.130826 1.949367
H 1.766723 0.494334 1.351047

[M-NO2](H2O)
E = -718.823830
N -1.385762 -1.193091 0.395982
N -1.988021 -0.001647 0.185116
C -2.922233 -0.141264 -0.770468
N -2.976906 -1.392990 -1.189507
C -2.015422 -1.994696 -0.447241
C -1.521795 1.198700 0.843113
C -0.299349 1.831000 0.184860
O 0.238177 2.803517 0.678892
N 0.058839 1.321728 -1.048465
C 1.449616 1.043955 -1.268847
C 1.908615 -0.170449 -0.452682
O 3.266562 -0.377632 -0.749964
C 3.801456 -1.473115 -0.041346
O 0.462872 -0.840432 2.567231
H -3.528553 0.685462 -1.123939
H -1.248277 0.956616 1.876249
H -2.332564 1.936127 0.855020
H 2.033730 1.919521 -0.934914
H 1.623729 0.872938 -2.335982
H 1.308503 -1.056638 -0.726145
H 1.763806 0.016954 0.625027
H 4.857329 -1.554233 -0.320003
H 3.282780 -2.410750 -0.307790
H 3.719320 -1.321314 1.047868
H 0.312544 -1.393327 3.336536
H -0.122554 -1.188664 1.872617
H -1.773053 -3.049096 -0.520029

[M-NO2](H2O)2
E = -795.240343
N 2.505308 0.601114 -0.740461
N 1.431201 -0.194043 -0.946122
C 1.517056 -1.293803 -0.174955
N 2.623957 -1.256382 0.547472
C 3.189771 -0.079753 0.162120
C 0.344864 0.191888 -1.815025
C -0.593583 1.221107 -1.201460
O -1.751789 1.333435 -1.574257
N -0.037887 2.123270 -0.323457
C -0.594747 2.247097 0.995335
C -0.236078 1.072213 1.904346
O -0.887052 -0.090848 1.441230
C -0.630684 -1.226769 2.255520
O -3.134508 -0.759554 -0.145508
O -1.273138 -2.609419 -1.000725
H 0.752816 -2.065436 -0.191905
H 0.755408 0.611659 -2.741660
H -0.246701 -0.700904 -2.046372
H -0.224378 3.172658 1.450438
H -1.693902 2.303772 0.904314
H 0.854878 0.912860 1.914806
H -0.565199 1.316705 2.928754
H -1.083373 -2.086886 1.751842
H 0.451668 -1.393989 2.359801
H -1.082407 -1.095883 3.251395
H -2.029407 -2.046386 -0.736345
H -1.658283 -3.419466 -1.339623
H -2.523802 -0.390879 0.512539
H -3.108823 -0.099655 -0.850130
H 4.135756 0.283329 0.548913

[M-NO2](H2O)3
E = -871.650526
N -1.872252 1.009349 -0.890973
N -1.549355 0.868620 0.415837
C -2.658840 0.587001 1.120497
N -3.714020 0.541575 0.323987
C -3.179130 0.806238 -0.893055
C -0.178837 0.992658 0.855894
C 0.335636 -0.305688 1.473605
O -0.375315 -1.089459 2.070383
N 1.714103 -0.451371 1.429997
C 2.290686 -1.278859 0.399682
C 1.359417 -2.333027 -0.179925
O 0.307649 -1.666281 -0.854040
C -0.902812 -2.404790 -0.919831
O 0.445573 0.576922 -2.554626
O 2.755087 1.462415 -1.475561
O 2.589996 2.450539 1.199195
H -2.646124 0.418720 2.190411
H 0.427616 1.277864 -0.005736
H -0.071011 1.791938 1.599838
H 2.621522 -0.582713 -0.396873
H 3.202520 -1.741953 0.800492
H 1.918908 -2.975603 -0.878334
H 0.947563 -2.960315 0.625416
H -1.617168 -1.790266 -1.476921
H -0.753071 -3.361157 -1.443228
H -1.289298 -2.588965 0.093640
H -0.384380 0.928185 -2.186336
H 0.483422 -0.308144 -2.158483
H 1.921285 1.262809 -1.957940
H 3.328236 1.906617 -2.103103
H 2.742465 1.622021 1.664714
H 2.697945 2.212297 0.263640
H -3.760400 0.857167 -1.807145

[M-NO2](H2O)4
E = -948.062607
N -0.734504 0.055950 -2.264859
N 0.433483 0.025580 -1.583759
C 0.682472 1.222241 -1.035657
N -0.293944 2.065150 -1.338740
C -1.131742 1.301700 -2.091266
C 1.201540 -1.190685 -1.417067
C 0.628417 -2.056982 -0.302875
O 1.298451 -2.454760 0.640176
N -0.663248 -2.492036 -0.487789
C -1.552600 -2.503211 0.641541
C -2.558753 -1.356863 0.549103
O -1.907285 -0.133060 0.803518
C -2.808639 0.961153 0.797711
O 0.316236 0.999277 2.220251
O 2.758646 -0.248781 1.891140
O 3.714893 0.651032 -0.458732
O -0.450536 3.485405 1.054619
H 1.580395 1.427612 -0.462945
H 1.174577 -1.749243 -2.359412
H 2.230803 -0.911071 -1.167741
H -2.124270 -3.440749 0.616562
H -0.996149 -2.430775 1.588627
H -3.021085 -1.338059 -0.451230
H -3.345770 -1.532321 1.302759
H -2.231013 1.866411 1.005620
H -3.296468 1.054050 -0.185365
H -3.580020 0.831154 1.573992
H 0.147985 1.911047 1.925582
H -0.397045 0.479784 1.822454
H 1.926282 0.229166 2.082675
H 2.473394 -1.144013 1.668746
H 4.639756 0.902008 -0.450524
H 3.502857 0.359597 0.456851
H -0.427539 3.238110 0.107013
H -0.006773 4.332607 1.126015
H -2.049838 1.675160 -2.530368

[M-NO2](H2O)5
E = -1024.471489
N 1.564452 -1.930735 -0.466289
N 1.333403 -0.775438 -1.131237
C 2.459603 -0.049905 -1.171389
N 3.436909 -0.686693 -0.544698
C 2.834910 -1.830021 -0.128298
C 0.033659 -0.488027 -1.692682
C -0.487679 0.892584 -1.333808
O 0.171477 1.766249 -0.791013
N -1.742160 1.128601 -1.851374
C -2.675466 1.883378 -1.063888
C -3.468155 0.989831 -0.104782
O -2.633102 0.592762 0.954853
C -3.312503 -0.227754 1.893152
O 4.651004 1.435930 0.842539
O 1.946531 1.834977 1.553917
O -0.126916 0.101781 2.132086
O -0.737265 -2.425062 1.256561
O -2.586037 -2.125718 -0.708298
H 2.525966 0.923495 -1.641083
H -0.682486 -1.241569 -1.339911
H 0.062660 -0.555791 -2.788342
H -3.383597 2.371278 -1.743775
H -2.137092 2.639514 -0.469262
H -3.855432 0.104566 -0.632752
H -4.316180 1.584457 0.278216
H -2.588541 -0.499412 2.666817
H -3.691067 -1.136814 1.404662
H -4.148088 0.325998 2.352005
H 5.377975 1.305211 1.454331
H 4.516500 0.584277 0.385362
H 1.577580 2.056284 0.691197
H 2.904481 1.738212 1.411448
H 0.700956 0.609978 2.037007
H -0.797954 0.617062 1.666241
H -2.873704 -3.000678 -0.975604
H -2.005742 -2.271594 0.074159
H -0.505081 -1.564735 1.665506
H 0.039967 -2.602233 0.707238
H 3.338913 -2.605518 0.436738

[M-NO2](H2O), ring open
E = -718.828463
N -3.587843 2.316643 -0.011136
N -2.049818 -0.743045 0.419416

C -3.243093 -1.036093 -0.029455
N -4.158880 -0.085970 -0.289391
C -3.807646 1.170955 -0.118718
C -1.141086 -1.818950 0.655346
C 0.227191 -1.599162 -0.027113
O 0.931024 -2.572923 -0.246007
N 0.546533 -0.326185 -0.312157
C 1.832110 0.026501 -0.882287
C 2.885357 0.223562 0.199854
O 4.077177 0.635330 -0.425810
C 5.136730 0.798597 0.485448
O -0.569562 2.312540 0.270053
H -3.575411 -2.064619 -0.214019
H -0.918623 -1.843370 1.735194
H -1.505551 -2.811693 0.350796
H -0.075162 0.434773 -0.044889
H 2.150255 -0.768571 -1.565868
H 1.709630 0.954711 -1.452566
H 2.541947 0.986434 0.923541
H 3.037027 -0.725151 0.745164
H 6.013525 1.120800 -0.086573
H 4.898529 1.565002 1.244336
H 5.369918 -0.148843 1.002043
H -1.530442 2.426087 0.256712
H -0.212075 3.152302 -0.025331

[M-NO2](H2O)2, ring open
E = -795.242698
N -3.291774 2.194929 -0.858531
N -1.813351 -0.611007 0.523949
C -2.648000 -1.085685 -0.364390
N -3.426459 -0.274757 -1.109111
C -3.320339 1.025354 -0.935326
C -0.953584 -1.547357 1.194169
C 0.244840 -1.832612 0.248245
O 0.069085 -2.498826 -0.761734
N 1.408366 -1.293843 0.643567
C 2.581753 -1.336737 -0.209908
C 2.623862 -0.184324 -1.199319
O 2.784400 1.030858 -0.490141
C 2.725378 2.164288 -1.330700
O 1.529702 1.105642 2.147184
O -1.057903 1.972892 1.584370
H -2.760045 -2.155460 -0.561698
H -0.613200 -1.101481 2.134785
H -1.436703 -2.517382 1.382033
H 1.392670 -0.582906 1.383158
H 3.472842 -1.309921 0.429100
H 2.573407 -2.281429 -0.764954
H 3.469479 -0.326320 -1.895160
H 1.691271 -0.162239 -1.788789
H 2.870493 3.047107 -0.698997
H 3.520238 2.131716 -2.094089
H 1.746732 2.233532 -1.833927
H -1.434437 1.200502 1.129195
H -1.457744 2.730633 1.149943
H 0.669507 1.547353 2.022973
H 2.032275 1.336955 1.353245

[M-NO2](H2O)3, ring open
E = -871.654845
C 3.058816 1.881663 1.805339
O 2.960528 1.125034 0.613019
C 2.333468 1.845543 -0.433749
C 2.228747 0.964282 -1.667038
N 1.316348 -0.150754 -1.495768
C -0.012378 0.040746 -1.499756
O -0.564133 1.116171 -1.690894
C -0.860109 -1.231324 -1.245873
N -1.713224 -0.967285 -0.118676
C -2.819295 -0.321799 -0.357216
N -3.580777 0.001772 0.711947
C -4.692983 0.662274 0.504264
N -5.708234 1.247786 0.420268
O 2.206461 -1.430342 1.601295
O 2.025961 -2.804348 -0.669619
O -0.374378 -0.856522 2.401346
H -3.133837 -0.044239 -1.372255
H -0.218116 -2.089192 -1.022120
H -1.424991 -1.413460 -2.174008
H 1.683974 -1.083348 -1.301194
H 3.214833 0.556378 -1.917898
H 1.882763 1.586064 -2.502760
H 2.934715 2.738213 -0.679981
H 1.330133 2.178355 -0.123133
H 3.545320 1.246215 2.552662
H 3.665459 2.787555 1.644514
H 2.060513 2.173665 2.169467
H 1.322638 -1.293041 1.991346
H 2.447054 -0.565347 1.229781
H 2.141978 -2.448560 0.244989
H 2.748501 -3.415944 -0.819221
H -0.955161 -0.951470 1.621818
H -0.897659 -1.130717 3.156582

[M-NO2](H2O)41, ring open
E = -948.066356
C -2.410323 2.886431 1.333154
O -2.414230 1.538861 0.905490
C -3.352773 1.290944 -0.120858
C -3.288500 -0.165003 -0.540887
N -2.021704 -0.487082 -1.165140
C -1.317123 -1.595026 -0.866084
O -1.723071 -2.502491 -0.150675
C 0.074132 -1.688917 -1.507319
N 0.524443 -0.408155 -1.978788
C 1.604156 0.058207 -1.405423
N 2.006362 1.290572 -1.772345
C 3.046408 1.793413 -1.147062
N 3.947767 2.327600 -0.618030
O 0.031728 0.115541 1.193017
O 0.310782 -2.499841 1.921890
O 2.780992 -1.850857 1.087598
O 2.566691 0.808537 1.942393
H 2.158949 -0.513919 -0.647653
H -0.035232 -2.358738 -2.374306
H 0.735469 -2.162444 -0.768984
H -1.550113 0.203531 -1.736090
H -4.124031 -0.364666 -1.228289
H -3.406020 -0.820664 0.329383
H -3.150259 1.954600 -0.982699
H -4.371797 1.513929 0.239542

H -1.662366 2.972319 2.128228
H -2.142235 3.563747 0.505571
H -3.396626 3.174899 1.731019
H 0.798291 0.627673 1.508065
H -0.750703 0.683865 1.158350
H 0.066185 -1.555388 1.855505
H -0.332902 -2.919965 1.334395
H 3.459585 -2.453857 1.397443
H 1.921666 -2.223248 1.402939
H 3.206644 1.368355 1.491454
H 2.818425 -0.108880 1.725407

[M-NO2](H2O)5, ring open
E = -1024.476499
N -6.159642 0.763433 -1.005682
N -2.066155 0.525800 1.074826
C -3.258578 0.237456 0.642599
N -3.887941 1.181835 -0.097299
C -5.083343 0.910981 -0.556048
C -1.366433 -0.515901 1.782343
C -0.789771 -1.534184 0.770843
O -1.522621 -2.044924 -0.073703
N 0.511775 -1.805655 0.935585
C 1.237578 -2.728895 0.080866
C 2.543966 -2.119788 -0.393481
O 2.265097 -1.042639 -1.259473
C 3.399143 -0.244955 -1.554870
O -0.257811 -0.273367 -2.111166
O 0.180832 1.690157 -0.272905
O 2.461903 3.094163 -0.688548
O 4.121279 1.888713 1.130639
O 1.748706 0.554271 1.813764
H -3.751401 -0.721488 0.853334
H -0.565991 -0.065378 2.377048
H -2.031601 -1.098736 2.440597
H 1.065055 -1.136472 1.480134
H 1.461409 -3.654464 0.630996
H 0.598497 -2.993109 -0.769641
H 3.131881 -1.758959 0.468418
H 3.138840 -2.889419 -0.915177
H 3.054241 0.591835 -2.169489
H 3.849049 0.148638 -0.631368
H 4.150496 -0.827499 -2.112343
H 1.277668 0.971479 1.069562
H 2.647186 0.923109 1.757177
H -0.003186 1.041001 -0.990232
H -0.663726 1.757949 0.198432
H 4.512481 2.506866 1.751251
H 3.624915 2.436256 0.487947
H 0.616152 -0.671742 -1.954292
H -0.881750 -0.909875 -1.736234
H 1.562031 2.727566 -0.567572
H 2.347049 4.005273 -0.962942
